# Lack of methoxy-mycolates characterizes the geographically restricted lineage 7 of *Mycobacterium tuberculosis* complex

**DOI:** 10.1099/mgen.0.001011

**Published:** 2023-05-12

**Authors:** Elena Hailu, Daire Cantillon, Carlos Madrazo, Graham Rose, Paul R. Wheeler, Paul Golby, Bethlehem Adnew, Sebastien Gagneux, Abraham Aseffa, Stephen V. Gordon, Iñaki Comas, Douglas B. Young, Simon J. Waddell, Gerald Larrouy-Maumus, Stefan Berg

**Affiliations:** ^1^​ Armauer Hansen Research Institute, Addis Ababa, Ethiopia; ^2^​ Brighton and Sussex Centre for Global Health Research, Department of Global Health and Infection, Brighton and Sussex Medical School, University of Sussex, Falmer, UK; ^3^​ Biomedicine Institute of Valencia, Spanish Research Council (IBV-CSIC), Valencia, Spain; ^4^​ Francis Crick Institute, London, UK; ^5^​ Animal and Plant Health Agency, Weybridge, UK; ^6^​ Swiss Tropical and Public Health Institute, Allschwil, Switzerland; ^7^​ University of Basel, Basel, Switzerland; ^8^​ School of Veterinary Medicine, University College Dublin, Dublin, Ireland; ^9^​ MRC Centre for Molecular Bacteriology and Infection, Imperial College London, London, UK; ^‡^​ Present address: Department of Tropical Biology, Liverpool School of Tropical Medicine, Liverpool, UK; ^§^​ Present address: North Thames Genomic Laboratory Hub, Great Ormond Street Hospital for Children, London, UK; ^#^​ Present address: Bernhard Nocht Institute for Tropical Medicine, Hamburg, Germany

**Keywords:** cell wall, cord factor, genomics, lineage 7, methoxy-mycolates, *Mycobacterium tuberculosis*

## Abstract

Lineage 7 (L7) emerged in the phylogeny of the *Mycobacterium tuberculosis* complex (MTBC) subsequent to the branching of ‘ancient’ lineage 1 and prior to the Eurasian dispersal of ‘modern’ lineages 2, 3 and 4. In contrast to the major MTBC lineages, the current epidemiology suggests that prevalence of L7 is highly confined to the Ethiopian population, or when identified outside of Ethiopia, it has mainly been in patients of Ethiopian origin. To search for microbiological factors that may contribute to its restricted distribution, we compared the genome of L7 to the genomes of globally dispersed MTBC lineages. The frequency of predicted functional mutations in L7 was similar to that documented in other lineages. These include mutations characteristic of modern lineages – such as constitutive expression of nitrate reductase – as well as mutations in the VirS locus that are commonly found in ancient lineages. We also identified and characterized multiple lineage-specific mutations in L7 in biosynthesis pathways of cell wall lipids, including confirmed deficiency of methoxy-mycolic acids due to a stop-gain mutation in the *mmaA3* gene that encodes a methoxy-mycolic acid synthase. We show that the abolished biosynthesis of methoxy-mycolates of L7 alters the cell structure and colony morphology on selected growth media and impacts biofilm formation. The loss of these mycolic acid moieties may change the host–pathogen dynamic for L7 isolates, explaining the limited geographical distribution of L7 and contributing to further understanding the spread of MTBC lineages across the globe.

## Data Summary

All *Mycobacterium tuberculosis* genomes analysed in this work are available at the European Nucleotide Archive (ENA) under the accession numbers listed in Table S1 (available with the online version of this article).

**Impact Statement**

Global molecular epidemiology of tuberculosis has to date identified nine phylogenetic lineages among the human-adapted members of the *Mycobacterium tuberculosis* complex (MTBC). This study focuses on MTBC lineage 7 (L7) that appears geographically restricted to Ethiopia. Genomic analysis discovered a set of L7 mutations clustered in key cell wall loci, including genes associated with mycolic acid biosynthesis. Here, we show that a stop-gain mutation in the *mmaA3* gene, encoding a methoxy-mycolic acid synthase, abolishes the biosynthesis of methoxy-mycolates in L7, resulting in altered cell structure and colony morphology. Given the critical nature of the mycobacterial cell wall in host–pathogen interaction, the effects of these mutations may explain the geographical restriction of MTBC L7. Our study provides new insights into the fundamental biology of host–pathogen interactions and into the evolution and diversity of MTBC lineages.

## Introduction

Tuberculosis (TB) is one of the most devastating bacterial infectious diseases of humans, killing 1.5 million people per year globally [1]. TB in humans, livestock and wild animals is caused by members of the *Mycobacterium tuberculosis* complex (MTBC), with each member having an apparent host preference [2]. *M. tuberculosis* and *Mycobacterium africanum* are the main members of MTBC that have adapted to cause TB in humans, while zoonotic transmission from animal-adapted MTBC species such as *Mycobacterium bovis* and *Mycobacterium orygis* can also cause TB in humans [3, 4]. The members of MTBC are highly related to each other with >99.9 % identity and, due to no or negligible recombination [5, 6], the MTBC is considered as clonal and to have developed mainly through loss of genetic information (large sequence polymorphism; LSP) or through SNP [7].

World-wide molecular epidemiology has so far identified nine phylogenetic lineages (L1–L9) among the human-adapted members of the MTBC, with six major lineages responsible for the majority of cases globally. Two of these lineages, L5 and L6, belong to *M. africanum* and they are restricted to West Africa [8, 9], whereas four major lineages, L1–L4, belong to *M. tuberculosis sensu stricto* [10–13] and they are prevalent globally, but with different geographical frequency. While L3 and L4 are common globally, L1 and L2 are more restricted to Africa and Asia. Three additional human-adapted lineages, L7–L9, have been identified in Africa, but from a smaller number of patients; only a handful of strains have been described so far from L8 or L9, making it difficult to understand their prevalence in host populations [14, 15]. L7, however, has been identified in significantly higher frequency, but all studies up to date suggest that patients infected with L7 have been largely restricted to Ethiopia [12, 13]. A recent systematic review of the molecular epidemiology of *M. tuberculosis* in Ethiopia explored publications from the last 20 years covering nearly 4400 typed MTBC strains of which L7 comprised 3.4 %, while L1 (7.3 %), L3 (21.7 %) and L4 (62.7 %) were the dominant TB lineages in Ethiopia during the last two decades [16]. L7 might have been underrepresented in these figures as it was discovered as a new lineage only in 2012.

The establishment of a robust phylogeny for the MTBC has raised a series of fundamental questions concerning the impact of genetic diversity on important clinical and epidemiological features of TB. While the relevant studies to explore these are in their infancy, there are initial indications that pathogen diversity can have a significant influence on early progression to disease, on the anatomical site of disease, and on the emergence and spread of multidrug resistance [17–20]. There is also intriguing evidence of association of particular pathogen genotypes with particular ethnic groups [21, 22], which may be a critical aspect in the interpretation of results of genome-wide association studies to characterize the influence of host factors on disease susceptibility. The discovery of *M. tuberculosis* L7 and its apparent restricted prevalence to the Horn of Africa [12, 13] raised questions as to whether this restriction was due to historical events, including possible transmission bottlenecks, or instead whether it was a function of genetic features of this lineage that make it less transmissible or particularly adapted to a specific host population in the Horn of Africa. In fact, a study from Ethiopia found that patients infected with L7 presented at health centres with a delay when compared to other *M. tuberculosis* lineages [23]. This may indicate that L7 patients progress more slowly to clinical TB disease than other lineages infections.

Since the discovery of *M. tuberculosis* L7, several studies have explored differences between L7 and other lineages of *M. tuberculosis *, LSP and SNP profiles for L7 with unique genomic signatures [23–25]. In addition, comparative proteomics and protein acetylation analyses have been performed between *M. tuberculosis* L4 and L7 [26, 27]. Although differences between the two lineages were proposed, which could influence a wide variety of fundamental cell processes including virulence, no individual gene of L7 was linked with a phenotype that can explain the restricted epidemiology of L7.

To exhaust whether there are genetic differences between L7 and the more successful MTBC lineages that can shed light on its geographical restriction and the potentially weaker nature of L7, we performed additional bioinformatics analysis on all L7 genomes published up to date (Table S1), by mapping them against the genome of an inferred common ancestor of the MTBC. Our analysis highlighted a set of SNPs conserved in genes of L7 that are clustered in three key cell wall loci of *M. tuberculosis *, including genes associated with mycolic acid biosynthesis. We used bioinformatic tools to predict phenotypic implications for these SNPs. Interestingly, we identified a previously unpublished stop-gain mutation in the *mmaA3* gene and could confirm that this mutation is strictly conserved in all L7 genomes available in public genome databases, including those genomes published by Yimer *et al*. [24]. This gene corresponds to *Rv0643c* in the *M. tuberculosis* H_37_Rv reference strain where it has been characterized as an enzyme (MmaA3) responsible for *O*-methylation of mycolic acids in the biosynthesis of methoxy-mycolic acids (mMAs), also known as methoxy-mycolates [28, 29]. mMA and the structurally related keto-mycolic acid (keto-MA) and α-mycolic acid (α-MA) were identified in mycobacteria in the 1970s, and it is generally agreed that mycolic acids are essential components for the potent biological activities of mycobacterial constituents such as trehalose mycolates (cord factor) [30], wax D (a peptide-glycolipid) [31, 32] and the cell wall skeleton (mycolic acid–arabinogalactan–peptidoglycan complex) [33]. Although the mycolic acid structures (reviewed by Brennan and Nikaido [34]) and the pathways for their biosynthesis (reviewed by Verschoor *et al*. and PaweŁczyk and Kremer [35, 36]) have been well characterized, the biological roles of the mycolic acids are not fully elucidated. However, it has been shown that the three main types of mycolic acid in *M. tuberculosis* play different and critical roles in host-interaction, e.g. in cholesterol accumulation, and could influence the virulence of this pathogen [37, 38]. In the present study, we investigated the phenotypic consequences of the stop-gain mutation of *mmaA3* and could show that it abolishes the biosynthesis of mMA and alters the cell structure and colony morphology in L7. We also discuss potential biological and epidemiological consequences of the lack of mMA in L7 strains of *M. tuberculosis *.

## Methods

### Mycobacterial strains and culturing

All *M. tuberculosis* strains used for phenotypic analyses in this study were clinical isolates from Ethiopian patients [12] and reference strain *M. tuberculosis* H_37_Rv. *M. tuberculosis* strains selected for genetic complementation were BTBH-211 (L7), BTBH-444 (L7), BTBH-568 (L7) and BTBH-273 (L3). All mycobacterial culturing media were based on mixed powder from Middlebrook 7H9 broth (Becton Dickinson), Middlebrook 7H10 agar (Becton Dickinson) and Middlebrook 7H11 agar (Becton Dickinson), and the following components were used for supplementation of the different culturing media (Table 1): sterile lysed ovine blood prepared from sheep blood defibrinated (TSC Biosciences), adult bovine serum (Gibco), OADC and ADC enrichments (Becton Dickinson), while glycerol, malachite green, fungizone, polymyxin B, trimethoprim lactate and amoxicillin were all sourced from Sigma-Aldrich.

**Table 1.** Mycobacterial growth media used in this study (see Table S4 for details of media composition)

Name of medium	Base	Additives	Reference
7H9 broth	Middlebrook 7H9	ADC; with or without glycerol	Becton Dickinson
7H10 agar	Middlebrook 7H10	OADC	Becton Dickinson
7H11 agar	Middlebrook 7H11	OADC	Becton Dickinson
Modified 7H11 agar	Middlebrook 7H11	OADC+supplements (see Table S4)	Gallagher and Horwill [88]

### Measurement of antimicrobial drug efficacy

The impact of mMA complementation on antimicrobial drug efficacy was determined by a colorimetric microbroth dilution assay [39]. Selected strains were cultured to log phase in Middlebrook 7H9 supplemented with ADC and 0.05 % Tween 80. Microtitre plates containing ranges (200 to 0.001 μg ml^−1^) of the hydrophilic anti-TB drug isoniazid or the hydrophobic anti-TB drug ofloxacin in duplicate were inoculated with cultures diluted to ~5×10^5^ c.f.u. ml^−1^, alongside no drug and no bacteria controls. The plates were incubated for 7 days at 37 °C. CellTiter-Blue (Promega) was added at a final concentration of 10 % (v/v) and incubated overnight at 37 °C. The minimum inhibitory concentration (MIC) was estimated to be the lowest dilution of drug where a colour change was not observed, comparing *mmaA3* complemented to empty vector (EV) and wild-type (WT) for each strain.

### Bioinformatics analysis

For this part of the study, we used 65 genomes of MTBC from the European Nucleotide Archive (ENA) database (Table S1), of which 52 were of *M. tuberculosis* L7, 12 were *M. tuberculosis* L1 to L6 and 1 genome was *M. bovis *. Genomic analysis was performed following a validated and published pipeline [40]. These 65 read files were pre-processed using fastp [41] to scan reads and trim low-quality ends with a mean window quality <20. Next, Kraken was used to taxonomically sort and filter the unique MTBC reads [42]. Filtered reads were mapped with bwa [43] to a predicted MTBC ancestor reference sequence [44] using default parameters, and then processed using the SAMtools [45] and Picard [46] programs. Variant calling was performed with VarScan [47] following published parameters [48]. Once variants had been obtained, we used a mapping filter to discard variants present in repetitive genomic regions such as in PE/PPE gene families or phages [5]. SNPs were annotated as nonsynonymous or synonymous using SnpEff [49].

### Phylogenetic analyses and identification of L7 SNPs

To build a phylogeny, a concatenated alignment was created with the fixed-SNP of all 65 MTBC genomes. This alignment consisted of 8892 non-redundant positions. We reconstructed a maximum-likelihood phylogeny with the 65 samples using the iq-tree 1.6.11 [50] software ‘-m GTR -nt 20 -vv -bb 1000’. The tree was visualized in iTOL v. 418. The same alignment of SNPs from the 65 MTBC strains was imported into Mesquite 3.70 [51], in parallel with the phylogenetic tree generated from the same data in iq-tree 1.6.11. We used the ‘Trace All Character’ option of the Mesquite suite to map polymorphisms to branches. The dataset of reconstructed positions was exported, and polymorphisms mapped to the L7 common branch were extracted.

### Predicting the functional effect of mutations

We used the Sorting Intolerant From Tolerant (sift) algorithm to predict nonsynonymous SNPs likely to affect protein function based on sequence homology [52, 53] (01/04/2022 from https://sift.bii.a-star.edu.sg). Briefly, sift looks for homologues in other bacteria of the gene of interest and (i) scores the conservation of the positions where mutations are found and (ii) weights this score by the nature of the amino acid change. These measures are incorporated into a normalized probability score, and a sift score ≤0.05 indicates predicted functional impact. The recommended >3.5 conservation threshold was used to filter out biased predictions, as this indicates predictions based on less diverse sequence alignments, which can lead to increased false positives. Analyses of conserved domains and predictions were performed at the Conserved Domains Database at NCBI [54] (https://www.ncbi.nlm.nih.gov/Structure/cdd/wrpsb.cgi; 01/04/2022) and alignment of protein sequences was performed by ClustalW [55].

### Genetic complementation

Selected *M. tuberculosis* strains were genetically modified, introducing the *M. tuberculosis* H_37_Rv *mmaA3* gene into the chromosomal *attB* site. A 1.2 kb DNA fragment containing the *mmaA3* gene flanked by the 3′ end of *mmaA2* and the 5′ end of *mmaA4* was PCR amplified using *M. tuberculosis* H_37_Rv chromosomal DNA and the primers mmaA3_f (5′-ccc**aagctt**accggctgcgccaagctgtt-3′; *Hin*dIII site in bold) and mmaA3_r (5′-ccc**aagctt**gtcttggttgggctaatcgg-3′; *Hin*dIII site in bold). The PCR product was digested with *Hin*dIII and cloned into the *Hin*dIII-digested integrating shuttle vector pKINT to generate the construct pPG114. The sequence of the cloned insert was verified by standard dideoxy sequencing methodology using the *mmaA3* cloning primers mmaA3_f and mmaA3_r, and the *mmaA3* internal annealing primers mmaA3_i_f (5′-accggctcgctgaagttggc-3′) and mmaA3_i_r (5′-cgctcgcaccgggtactgct-3′). pPG114 was electroporated into the selected *M. tuberculosis* strains, which were then plated onto solid 7H11 medium containing 0.5 % (v/v) glycerol, OADC, 2 % sucrose and kanamycin (50 µg ml^−1^). Kanamycin-resistant transformants containing pPG114 integrated into the *attB* chromosomal site were selected after 4–6 weeks incubation at 37 °C. Transformants of negative (EV) controls were selected using the same procedure but with a pKINT vector lacking the *mmaA3* gene sequence.

### Lipid extractions and TLC

Mycobacterial cells were harvested from *M. tuberculosis* cultures grown to mid or late log phase, washed with sterile water, heat inactivated at 80 °C for 1 h and lyophilized. For preparation of radiolabelled lipids, the protocol described by Wheeler *et al*. [56] was used with the exception that 1 mCi sodium ^3^H-acetate (American Radiolabelled Chemicals; specificity 75–150 mCi mmol^−1^) was added per 100 ml culture medium. A minimum of 2 mg lyophilized cells of each strain was used for extraction of lipids following previously described protocols [57]. In short, lipids in the cell envelope that were not covalently bound to the cell wall (‘unbound lipids’) were extracted from the freeze-dried bacteria in a series of extractions. The residual pellet after the chloroform/methanol/water extractions was essentially cell wall mycolyl–arabinogalactan–peptidoglycan (mAGP). The pellet was washed in 1 ml chloroform/methanol (2 : 1, v/v), dried, saponified in 1 ml 15 % (w/v) tetrabutylammonium hydroxide for 18 h at 105 °C, cooled, then 2 ml water and 0.25 ml iodomethane were added along with 2 ml dichloromethane, mixing for 30 min to methylate the mycolic acids. The lower phase, containing methyl-mycolates, was then kept and washed twice with 0.1 M HCl and dried. This was further washed by dissolving it in hexane, washing with 0.1 M HCl and drying. The methyl-mycolates were then separated on TLC plates developed three times in hexane/ethyl acetate (95 : 5, v/v) [58, 59]. Developed TLC plates with radiolabelled mycolic acid products were visualized by electronic autoradiography (Phosphorimager), while plates with non-radiolabelled products were detected by spraying with 5 % ethanolic molybdophosphoric acid (MPA) followed by heating at 180 °C for 15 min.

### Colony and cell morphology imaging

To facilitate safe imaging of colonies, 7H10 and 7H11 agar plates were formaldehyde treated for 24 h to kill *M. tuberculosis *. Such treatment had no impact on colony morphology. Images were captured with a digital camera from a minimum of four independent experiments. For analysis by transmission electron microscopy (TEM), mycobacterial strains were cultured for 5–7 days to mid log phase (10^6^ to 10^8^ c.f.u. ml^−1^) in 7H9 liquid medium supplemented with 0.5 g fraction V BSA l^−1^, 0.05 % tyloxapol, 0.2 % dextrose, 0.2 % (v/v) glycerol and 10 mM NaCl. Cells were then washed three times in distilled water (17 000 ***g*** for 2 min) and suspended in a final volume of 50 µl. For negative staining, 10 µl washed mycobacteria preparations were applied to collodion-carbon-coated copper grids (200 meshes). After 10 s, excess solution was blotted off with filter paper and samples were stained with 1 % (w/v) uranyl acetate for 30 s. Analysis was performed using a Jeol 120 EX transmission electron microscope and images were acquired with a digital camera (AMT) at ×2000–4000 magnification. The experiment was also performed in 7H9 medium (as described above) but with the addition of 2 % (v/v) glycerol.

### Biofilm model

*M. tuberculosis* biofilms were cultured as described elsewhere [60]. Briefly, *M. tuberculosis* strains were grown to mid log phase in 7H9 broth supplemented with ADC and 0.05 % Tween 80. The cultures were diluted 1 : 100 in Sauton’s medium (0.05 % KH_2_PO_4_, 0.05 % MgSO_4_, 0.4 % l-asparagine, 0.2 % citric acid, 0.005 % ferric ammonium citrate, 6 % (v/v) glycerol; adjusted to pH 7.0, autoclaved and then supplemented with 0.1 % sterile ZnSO_4_). This inoculum was dispensed in 4.5 ml volumes per well in a sterile 12-well plate (Thermo Scientific). The plate was sealed with parafilm and incubated statically for 5 weeks at 37 °C before imaging. The experiment was repeated in duplicate from independent biological replicates.

## Results

### Ethiopian L7 strain population

An earlier study by us [12] isolated 36 *M *. *tuberculosis* strains from Ethiopian TB patients with spoligotype patterns that diverged from any previously known pattern marked by loss of spoligotype spacers 4–24 and 28–29 in the direct repeat region [61]. The 36 strains were shown to belong to a new *M. tuberculosis* lineage that was named L7 [12, 13]. For this study, we used 25 *M *. *tuberculosis* L7 genomes previously described by us [62] and supplemented these with 27 genomes of L7 from a more recent study [24]; together they represent all L7 genomes available at that date (01/09/2022) at the ENA and all 52 genomes are clinical isolates with origin from the Ethiopian highlands.

### Bioinformatic analysis of L7

**Fig. 1.**
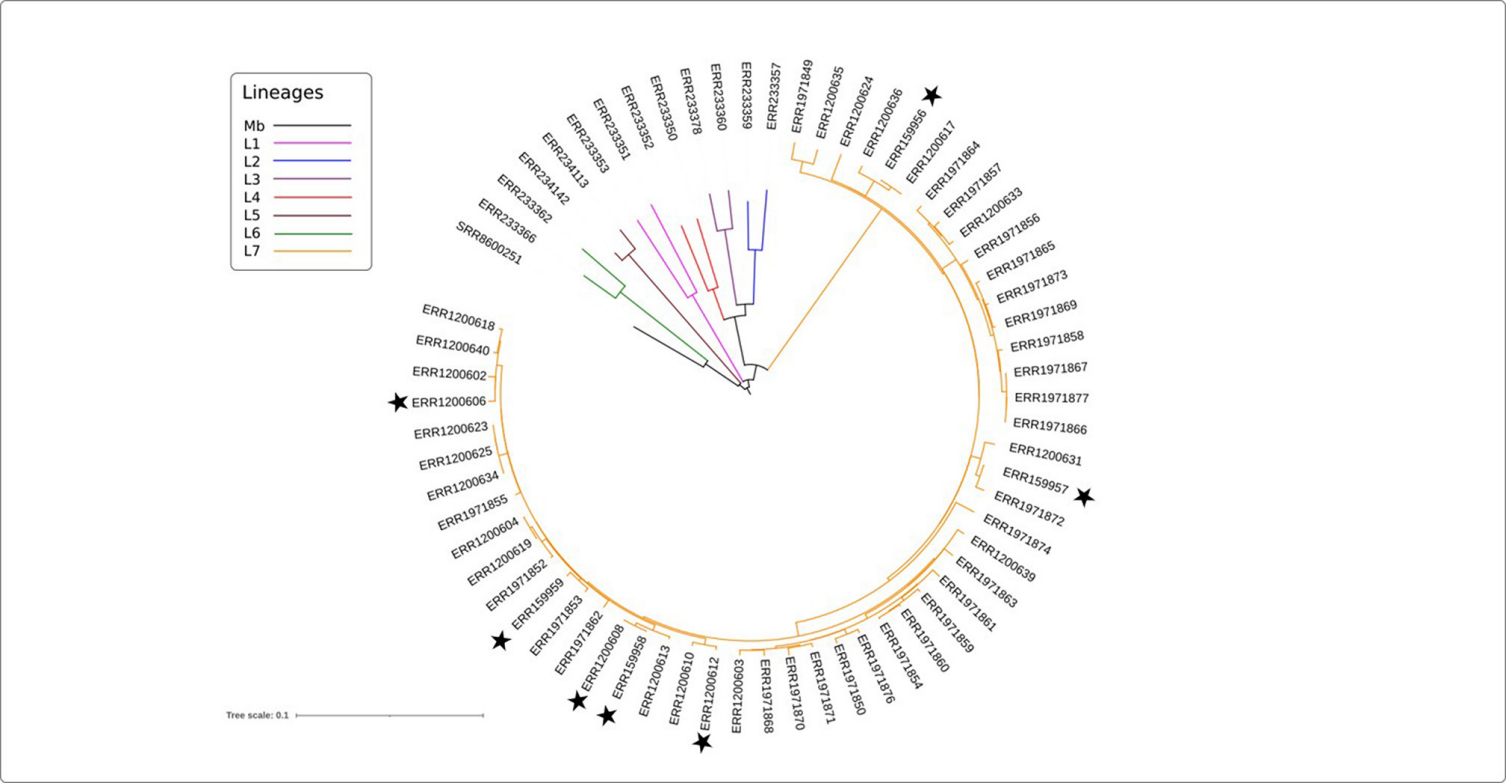
Maximum-likelihood phylogenetic tree of 65 MTBC genomes including 52 genomes of L7. Lineages are highlighted as follows: L1 in pink, L2 in blue, L3 in purple, L4 in red, L5 in brown, L6 green, L7 in orange and *M. bovis* (Mb) in black. The strain IDs for the respective ENA accession numbers are listed in Table S1. Accession numbers representing L7 strains used for the phenotypic experiments in this study are labelled with a black star.

We reconstructed a maximum-likelihood phylogeny (Fig. 1) using the 52 L7 genomes together with 12 genomes of *M. tuberculosis* L1 to L6, and 1 *M. bovis* genome (animal-adapted MTBC strain) from the ENA database (Table S1). Comparison of the L7 genome sequences with an inferred common ancestor allowed high confidence identification of 809 lineage-specific SNPs and a total number of 482 non-synonymous mutations (Table S2). The dN/dS ratio of the internal L7 branch was 0.64.

**Fig. 2.**
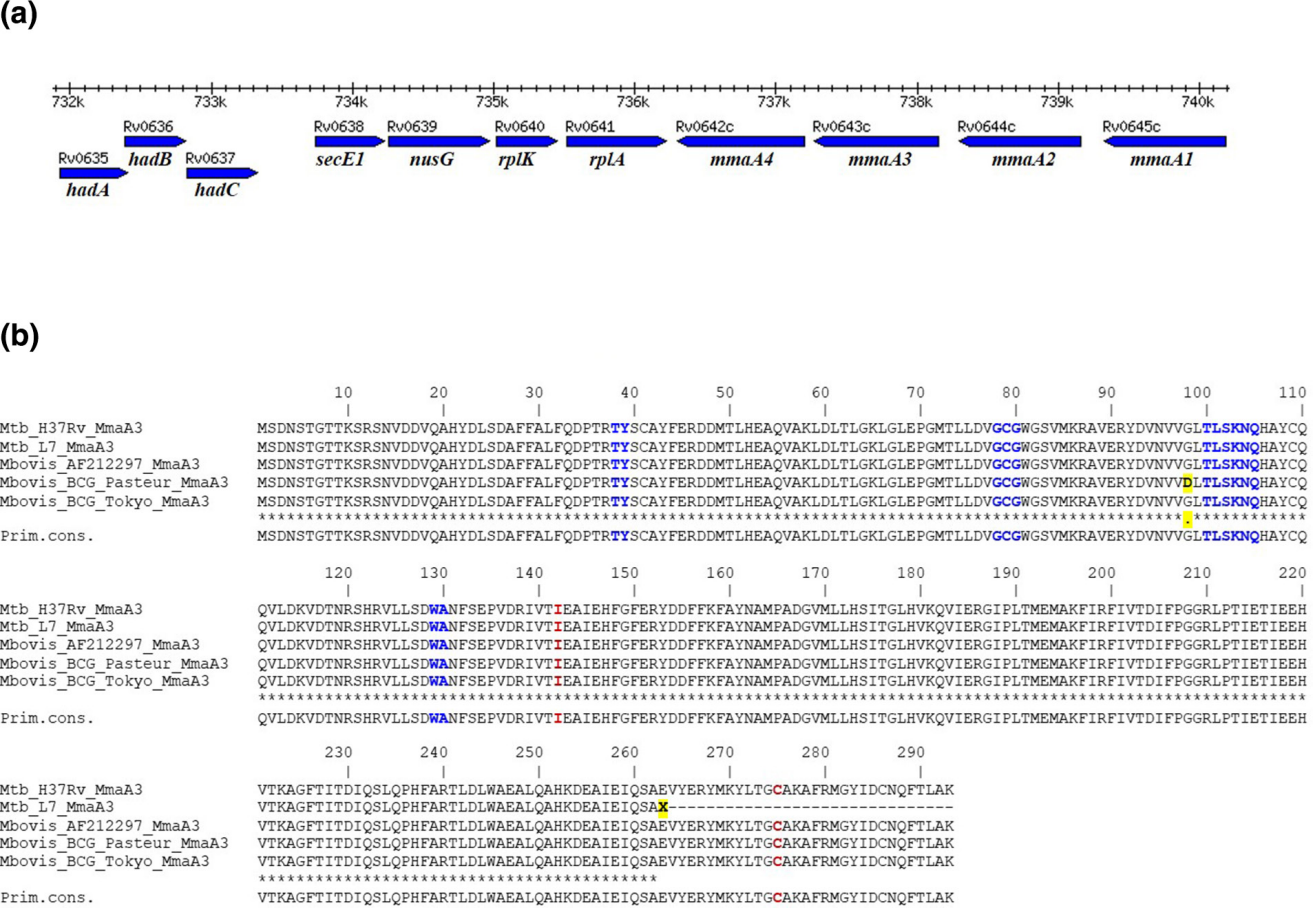
(a) Gene cluster *Rv0635–Rv0647c* of *M. tuberculosis *. This cluster includes the *hadABC* and *mmaA* genes that are involved in mycolic acid biosynthesis. (b) Amino acid sequence alignment of MmaA3. Sequences of MmaA3 from *M. tuberculosis* H_37_Rv (Rv0643c) and its homologues in *M. tuberculosis* L7, *M. bovis* AF2122/97 and two *M. bovis* BCG strains, BCG Pasteur and BCG Tokyo, were aligned using ClustalW (Expasy.org). Amino acid residues of MmaA3 homologous to a proposed SAM binding site are highlighted in blue, and residues predicted to be included in the active site are highlighted in red. Two point-mutations in this alignment of MmaA3 are highlighted on yellow backgrounds: the mutation explored in this study, E263X in L7, is a consequence of a stop-gain mutation (GCG→GAG) leading to a truncated protein, while the other mutation represents an evolutionary branch of *M. bovis* BCG (including BCG Pasteur) that carries point mutation G98D.

A sift analysis was performed on all non-synonymous SNPs and the results are listed in Table S3. The screening for functional SNPs using the sift algorithm identified 116 genes with mutations that are predicted to have an impact on protein function, either through exchange of amino acid, a stop-gain or a stop-loss mutation, corresponding to 13 % of the lineage-specific SNPs. More than half of the affected genes are predicted to play a role in transport or in metabolism of lipids and cell wall components. Among the 116 genes with SNPs predicted to have functional impacts on the expressed proteins, we identified 16 affected genes clustered in three key cell wall loci of L7 (Table 2, Fig. 2a) that are involved in biosynthesis of phthiocerol dimycocerosate (PDIM), phenolic glycolipid (PGL) and mycolic acids, which are lipid components highly important for the cell wall structure of *M. tuberculosis* but also as virulence factors. The location of the non-synonymous SNPs in relationship to predicted conserved protein domains are shown in Fig. S1(A–I) for the following selected genes: *Rv3080c* (*pknK*), *Rv3089* (*fadD13*), *Rv2931* (*ppsA*), *Rv2940c* (*mas*), *Rv2946c* (*pks1*), *Rv2947c* (*pks15*), *Rv2952*, *Rv2962c* and *Rv0643c* (*mmaA3*).

**Table 2.** Selected genes of *M. tuberculosis* L7 with conserved non-synonymous SNPs clustered in three cell wall loci Protein mutations that were predicted by sift as having functional impact are highlighted in bold. X, Stop-gain mutation.

Cluster	Gene	nsSNP in L7
PDIM/PGL	***Rv2931* (*ppsA*)**	**V728M**
	*Rv2935* (*ppsE*)	K1342Q
	*Rv2938* (*drrC*)	T255S
	***Rv2940c (mas)***	**V73F**
	***Rv2946c* (*pks1*)**	**A1437V**
	***Rv2946c* (*pks1*)**	**P1151S**
	***Rv2947c* (*pks15*)**	**S228P**
	***Rv2952***	**R184H**
	*Rv2958c*	F409L
	*Rv2962c*	E132A
VirS/MymA	***Rv3080c (pknK)***	**L237R**
	***Rv3083 (mymA)***	**G13S**
	***Rv3084 (lipR)***	**S198X**
	***Rv3089 (fadD13)***	**A43S**
	***Rv3089 (fadD13)***	**K172T**
Mycolic acid modification	***Rv0643c (mmaA3)***	**E263X**

In the PGL/PDIM locus (Table 2), referred to as the *Rv2930–Rv2962c* genes in *M. tuberculosis* H_37_Rv [63], there is a characteristic 7 bp deletion (GCCGCGG) in the *pks15/1* region [64] in L4 of *M. tuberculosis* that leads to a frameshift and absence of PGL production. Here, we could identify this sequence as present in 29 of the 52 L7 samples. In the remaining 23 L7 genomes, we were not able to confirm this 7 bp sequence as present, but it was likely due to low sample depth coverage (mean depth range was 30 to 300) as the very strong correlation shows in Fig. S2. Its presence in L7 suggests that the two ORFs of *pks15* and *pks1* are intact, a precondition for expression of the PGL-associated polyketide synthase and thereby PGL synthesis. However, L7 strains have three predicted functional SNPs in the *pks1* and *pks15* genes (Table 2) suggesting that, despite intact ORFs, their function in PGL synthesis could still be impaired. Other predicted functional SNPs in the PGL/PDIM locus were also identified: mutation R184H in methyltransferase Rv2952 is close to the previously documented G176R mutation [63]. The non-synonymous SNP of *ppsA* seems not to be in the region of the predicted active site (Fig. S1C), but may instead have structural impact on the protein and its function. A L7 SNP in glycosyltransferase Rv2962c (E132A) is predicted by sift analysis to be tolerated, and a lineage SNP in the MmpL7 transporter (L534V, Rv2942) was not scored by sift due to insufficient homology (Table S3).

Proteins of the MymA locus (*Rv3083–Rv3089*; Table 2) are involved in mycolic acid biosynthesis and are transcriptionally regulated by VirS [65]. L7 has predicted functional SNPs in the PknK regulator (Rv3080c), and in the monooxygenase (Rv3083) and Acyl-CoA synthase (Rv3089) proteins of the MymA locus, including a stop codon in LipR (Rv3084), a probable *N*-acetyl-hydrolase/esterase, leading to a truncation of 110 aa and a likely loss of function.

The third key cell wall gene cluster that we investigated for functional SNPs (*Rv0635–Rv0647c*; Fig. 2a) includes two operons that are involved in modifications of unsaturated mycolic acid chains: the *hadA-hadB-hadC* genes of the fatty acid synthase II (FAS II) operon and the *mmaA1-mmaA2-mmaA3-mmaA4* genes. Besides a SNP in *hadB* that was predicted by sift as tolerated, the only mutation identified as L7 specific in this gene cluster was a stop-gain mutation Glu263Stop (G787T) in the *mmaA3* gene (Table S3). In addition, we looked for other potential stop codons in the *mmaA3* region in a data set of 4958 *M *. *tuberculosis* isolates [66]. We identified two other substitutions that potentially caused stop codons: Glu53Stop (G157T in a L1 single isolate) and Trp129Stop (G386A in a L4 single isolate). Furthermore, we found another synonymous position that kept the pre-existing stop-codon: Stop294Stop (G882A; in a L1 single isolate).

### Analysis of the stop-gain mutation in *mmaA3*

From the above bioinformatics analysis of *M. tuberculosis* L7, we decided to perform more in-depth analysis of the consequences of the stop-gain mutation identified in codon 263 of the *mmaA3* gene. This mutation leads to a truncated 789 bp long ORF that, if expressed, can be translated to a protein with 262 aa as compared to 293 aa of the full-length wild-type protein. The sequential effect at the amino acid level is shown in Fig. 2(b), in an alignment between MmaA3 (Rv0643c) of the reference strain *M. tuberculosis* H_37_Rv and its respective homologue in strains of *M. tuberculosis* L7. MmaA3 of *M. tuberculosis* H_37_Rv is a functional protein [28] and shares 100 % identity with homologues in all defined lineages of the MTBC. A Conserved Domain analysis was performed on MmaA3; the result shown in Fig. S1 highlights several conserved domains including a proposed *S*-adenosylmethionine (SAM) binding site. Conserved residues of the SAM binding site [67] are marked in the sequence alignment of MmaA3 in Fig. 2(b). The next step was to investigate whether a truncated MmaA3 protein also had an impact on the cell wall phenotype of L7.

### Lipid analysis – mycolic acid modification

**Fig. 3.**
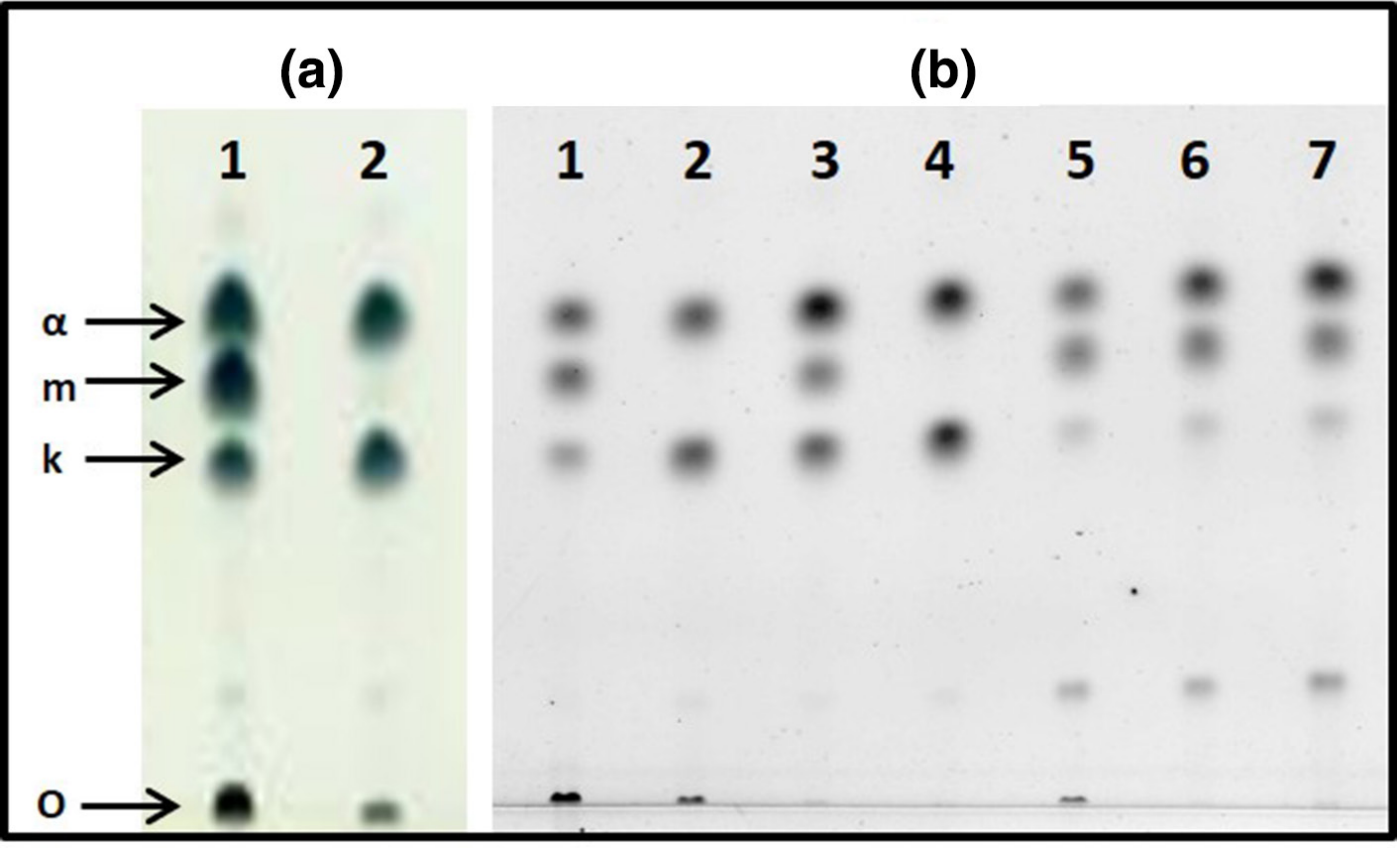
TLC profiles of mycolic acids from *M. tuberculosis *. Lipid fractions containing mycolic acids extracted from the mAGP of selected *M. tuberculosis* strains were methylated and separated on TLC plates developed three times in hexane:ethyl acetate (95 : 5). Lipids were detected by molybdophosphoric acid (**a**) or by electronic autoradiography (**b**). The product application origin (o) as well as α- (α), methoxy- (m) and keto (k)-mycolic acids are shown by arrows. (a) Mycolic acid profiles of *M. tuberculosis* H_37_Rv of L4 (lane 1) and *M. tuberculosis* BTBH-211 of L7 (lane 2). (b) Mycolic acid profiles of reference strain *M. tuberculosis* H_37_Rv of L4 (lane 1), and selected wild-type and mutant isolates of L7: BTBH-211 wild-type (lane 2), BTBH-211::mmaA3 (lane 3), BTBH-211 EV control (lane 4), BTBH-273 wild-type (lane 5), BTBH-273::mmaA3 (lane 6), BTBH-273 EV control (lane 7).

Knowing that MmaA3 had been characterized as an enzyme involved in the biosynthesis of mMA [28] encouraged us to explore whether a truncated MmaA3 in L7 has an impact on its mycolic acid biosynthesis. Targeting the composition of mycolic acids of the mAGP in the cell envelope, a series of lipid extractions were performed on 13 different *M. tuberculosis* strains; seven clinical isolates of *M. tuberculosis* L7 (strain IDs BTBH-122, BTBH-127, BTBH-211, BTBH-217, BTBH-444, BTBH-500 and BTBH-568), two clinical isolates of *M. tuberculosis* L3 (strain IDs BTBH-273 and BTBH-463), three clinical isolates of *M. tuberculosis* L4 (strain IDs BTBH-485, BTBH-587 and BTBH-721), as well as the reference strain *M. tuberculosis* H_37_Rv of L4. The seven selected L7 strains in this experiment represented a wide range of sub-lineages of L7 (highlighted in Fig. 1, Table S1) and included the two most common L7 spoligotypes in our strain collection, SIT910 and SIT1729 [12]. After extraction of non-covalently bound (polar and non-polar) lipids according to the classical protocols by Dobson *et al*. [57], the remaining pellet, containing cell wall mAGP, was hydrolysed and methylated to yield methyl mycolates. The mycolate composition of these strains was analysed by TLC. All investigated isolates of L3 and L4 (including H_37_Rv) could be identified with α-, methoxy- and keto-mycolates, the three common mycolic acids of *M. tuberculosis *, while all L7 isolates showed the presence of α- and keto-mycolates only (Fig. 3). We concluded that all examined strains of L7 lacked methoxy-mycolates, while they were present in the investigated strains of L3 and L4.

### Complementation of MmaA3 restored wild-type phenotype

Three L7 strains (BTBH-211, BTBH-568 and BTBH-444) and one L3 strain (BTBH-273) were genetically modified by introducing the *mmaA3* gene (from *M. tuberculosis* H_37_Rv) into the chromosomal *attB* site [68]. Successful genetic complementation of the selected isolates (as shown by Kan^R^ and confirmation by sequencing across the modified *attB* site) generated tools to explore the phenotypic effect of the MmaA3 truncation. Complementation and expression of full-length *mmaA3* in these L7 strains restored the production of mMA. In comparison, the strains transformed with an EV control, containing no full-length *mmaA3* sequence, showed the same mycolic acid profile as the wild-type L7 strains. Representative mycolic acid profiles of these genetic constructs of isolate L7 BTBH-211 are shown in Fig. 3(b). Therefore, we concluded that the stop-gain mutation E263X in MmaA3 of L7 abolishes its enzymatic activity and the biosynthesis of mMA, and that this activity could be restored by complementation with the *mmaA3* gene of *M. tuberculosis* H_37_Rv. We also investigated the outcome of over-expressing the MmaA3 protein in BTBH-273::mmaA3. As expected, all three types of mycolic acids were present in the corresponding genetic constructs of BTBH-273 (L3). However, the ratios of abundancy between the three lipids were not investigated in the three constructs. In the work that followed this discovery of L7 strains lacking mMA, we explored different consequences of this phenotype.

### *In vitro* growth and drug susceptibility

To understand whether the lack of mMA would have an impact on *in vitro* growth, six L7 strains (BTBH-568 wild-type, BTBH-568::mmaA3 and BTBH-568 EV control; BTBH-444 wild-type, BTBH-444::mmaA3 and BTBH-444 EV control), three L3 strains (BTBH-273 wild-type, BTBH-273::mmaA3 and BTBH-273 EV control) and the reference strain *M. tuberculosis* H_37_Rv were cultured in Middlebrook 7H9 supplemented with ADC and 0.05 % Tween 80 (Table S4). Optical density values of these 10 cultures were measured over 23 days, followed by serial dilution and plating on both 7H10 and 7H11 agar plates to estimate c.f.u. counts. No significant differences in growth characteristics were observed between strains, showing no correlation with mMA expression (Fig. S3). This suggested that the absence of mMA does not influence axenic growth in nutrient-replete liquid media.

The same 10 *M *. *tuberculosis* strains were also used to assess whether changes to the cell wall structure (due to lack of mMA) would have an impact on the efficacy of hydrophilic or hydrophobic antimicrobial drugs, isoniazid and ofloxacin, respectively. Using an established colorimetric assay, the expression of mMA did not change the MIC of these two drugs in the L7 or L3 strains.

### Change of cell morphology in liquid culture

**Fig. 4.**
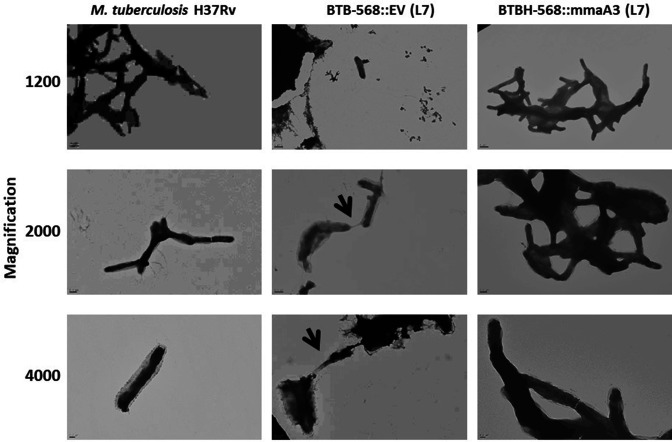
TEM structures of L7 grown in 7H9 broth. L7 strain BTBH-568 complemented with *mmaA3* and EV control in comparison to *M. tuberculosis* H_37_Rv. A Jeol 120 EX transmission electron microscope was used for analysis and images were acquired at ×1200–4000 magnification. Arrows highlight the reduced cording phenotype of L7, suggesting an impaired cell envelope.

The effect of L7 being deficient in mMA was explored at the cellular level by TEM. *M. tuberculosis* H_37_Rv (L4) and strain BTBH-568 of L7 (the EV control and *mmaA3* complemented strains) were cultured in liquid Middlebrook 7H9 and their phenotypes were observed by TEM. Although the growth rates were similar for the three strains, the L7 wild-type strain formed a reduced cording phenotype, and the bacterial cells were not well separated and appeared to secrete/shed material into the medium, suggesting an impaired cell envelope. In comparison, both the *mmaA3* complemented L7 strain and the reference strain H_37_Rv showed well-defined cell structures as expected for *M. tuberculosis* (Fig. 4).

### Complementation with *mmaA3* altered biofilm growth

**Fig. 5.**
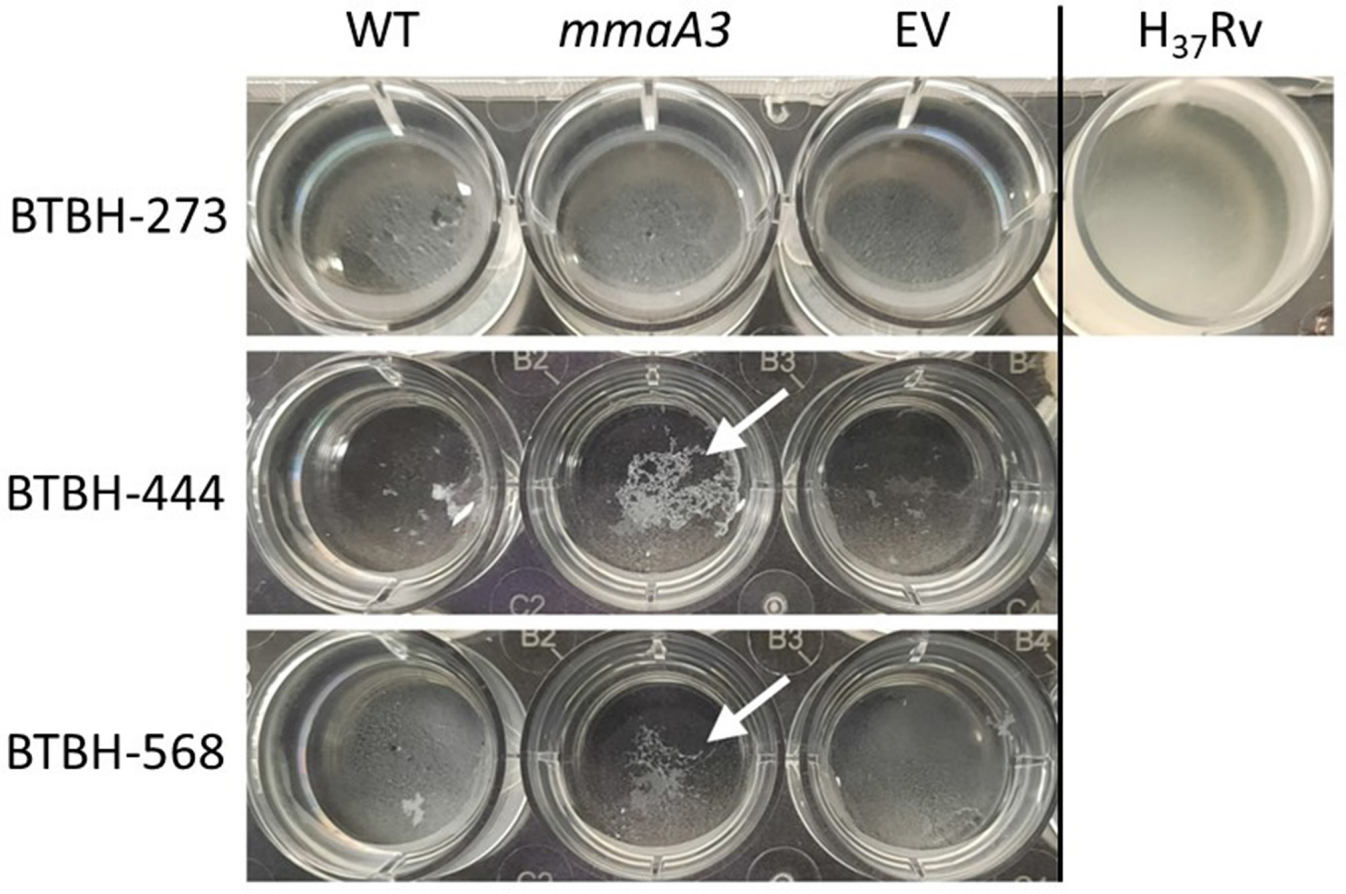
Growth of *M. tuberculosis* biofilms. Representative images of *M. tuberculosis* biofilms after 5 weeks incubation in 12-well plates are shown for BTBH-273 wild-type (L3), BTBH-273::mmaA3 (L3), BTBH-273 EV control (L3); BTBH-444 wild-type (L7), BTB-444::mmaA3 (L7), BTBH-444 EV control (L7); BTBH-568 wild-type (L7), BTBH-568::mmaA3 (L7), BTBH-568 EV control (L7); and for *M. tuberculosis* H_37_Rv (L4). Only *M. tuberculosis* H_37_Rv formed a surface attached pellicle. BTBH-273, BTBH-444 and BTBH-568 grew biomass on the well bottom to varying extents. *mmaA3* complementation had no impact on L3 BTBH-273 biofilm growth. For L7 BTBH-444 and BTBH-568, *mmaA3* complementation led to a more knotted, corded growth phenotype (highlighted by arrows). This experiment was repeated in duplicate, as independent biological replicates.

Strains were subsequently assessed to determine whether the reduced cording seen in the L7 wild-type strains impacted biofilm formation. *M. tuberculosis* H_37_Rv formed a surface pellicle as expected, but none of the investigated strains BTBH-273 (L3), BTBH-444 (L7) and BTBH-568 (L7) formed surface pellicles (Fig. 5). These three clinical strains did, however, grow as a layer of biomass on the well bottom. *mmaA3* complementation of strain BTBH-273 (of L3 with intact *mmaA3*) did not affect the growth phenotype, but in the two L7 strains BTBH-444 and BTBH-568, *mmaA3* complementation lead to a different growth phenotype compared to their respective wild-type and EV control strains. While the L7 wild-type and the EV control strains had biomass grown as a homogenous layer with minimal cell clumps, the L7 complemented strains appeared macroscopically as dense, corded knots or aggregates, suggestive of increased cording formation on expression of active MmaA3.

### Strains of L7 presented an abnormal colony morphology on modified 7H11 agar

**Fig. 6.**
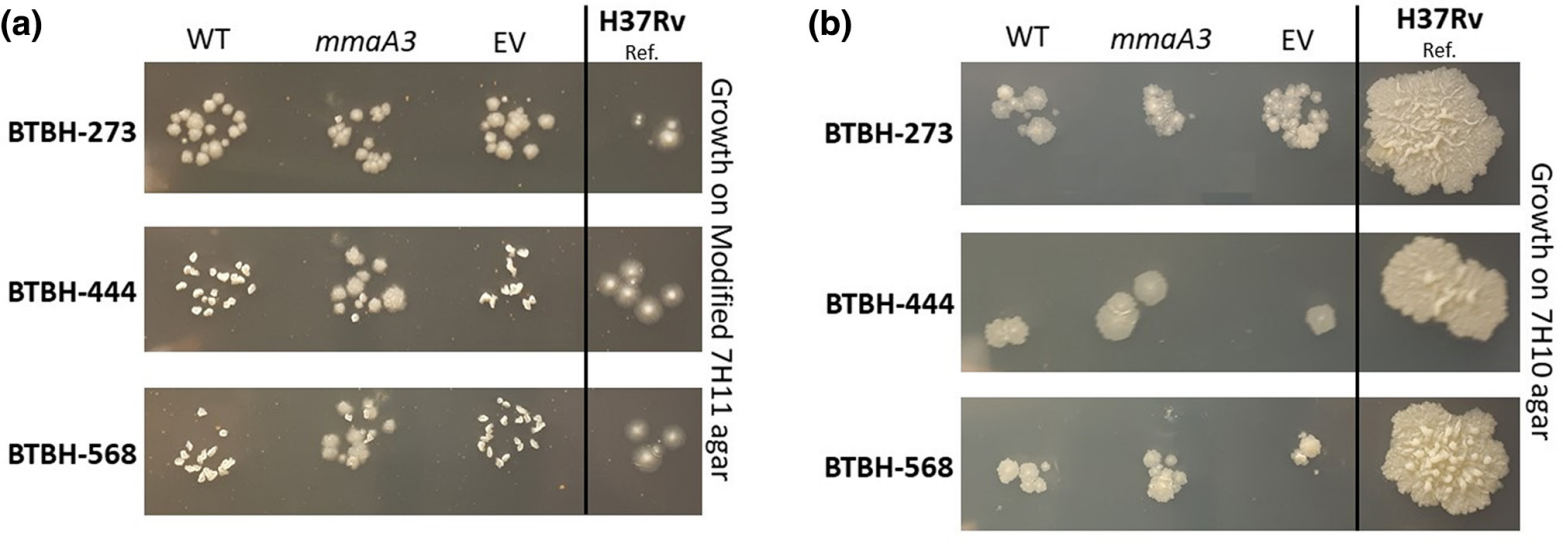
Colony morphology of *M. tuberculosis *. Representative colony images of L7 and L3 strains grown for 5 weeks, showing a difference in L7 colony morphology on (**a**) modified 7H11 agar medium, but not on (**b**) 7H10 agar medium. Images of colonies are shown for BTBH-273 wild-type (L3), BTBH-273::mmaA3 (L3), BTBH-273 EV control (L3); BTBH-444 wild-type (L7), BTBH-444::mmaA3 (L7), BTBH-444 EV control (L7); BTBH-568 wild-type (L7), BTBH-568::mmaA3 (L7), BTBH-568 EV control (L7); and for *M. tuberculosis* H_37_Rv (L4). This experiment was repeated a minimum of four times from independent biological replicates.

The colony morphology of *M. tuberculosis* grown on Middlebrook agar medium has a classical structural feature referred to as eugonic [69]. However, when wild-type isolates of *M. tuberculosis* L7 were grown on modified 7H11 agar (Fig. 6a, Table S4), visual inspection revealed a distinct difference in colony morphology when compared to the reference strain *M. tuberculosis* H_37_Rv, as well as strains of other *M. tuberculosis* lineages. Instead of a flat structural feature, L7 wild-type isolates had a raised crumpled morphology when grown on this medium. To explore whether the difference in morphology was associated with the absence or presence of mMA, we grew *M. tuberculosis* L7 (BTBH-444 and BTBH-568), *M. tuberculosis* L3 (BTBH-273) and their respective constructs complemented with *mmaA3* or EV control on modified 7H11 agar. The result showed that genetic complementation of the L7 isolates with *mmaA3* reverted colony morphology towards the classical features of other *M. tuberculosis* lineages (Fig. 6a), while the wild-type strains and their EV controls retained their original raised crumpled morphological features. As a comparison, the corresponding constructs of *M. tuberculosis* BTBH-273 (L3) showed no differences in colony morphology on modified 7H11 (Fig. 6a), even when MmaA3 was overexpressed (BTBH-273::mmaA3). We concluded that this conversion of the colony morphology for L7 on modified 7H11 agar, when complemented with full-length *mmaA3*, was due to restoration of methoxy-mycolate biosynthesis that had a direct or indirect effect on cell wall structure.

It can be noted that the distinct morphology seen for L7 wild-type strains when cultured on modified 7H11 agar medium could be repeated on a range of other Middlebrook 7H11 agar media supplemented with different components. However, this colony morphology was not observed when the related Middlebrook 7H10 agar medium was used (Fig. 6b). Instead of raised and crumpled colony morphology for L7 wild-type strains, as seen on modified 7H11 agar, they presented with an eugonic colony morphology (and not raised from the solid medium) but still significantly smaller than the *mmaA3*-complemented L7 strains. Furthermore, the addition of glycerol to both 7H11 and 7H10 agar media had an impact on the morphology of the wild-type L7 strains, showing a more eugonic appearance with increased glycerol concentration. Glycerol also has an impact on cellular morphology as measured by TEM. The results from these experiments and the composition of respective culture media used are described in the Supplementary Material (supplementary text, Table S4, Figs S4–S6). In summary, the addition of glycerol or the absence of 0.1 % pancreatic digest of casein (present in Modified 7H11 but not in 7H10) is likely responsible for the restoration of the L7 morphology to eugonic when grown on these media *in vitro*, but additional experiments would be needed to fully explore their effects. The current findings, however, cannot clarify the possible role that mMA has in host–pathogen interactions and as a virulence factor *in vivo*.

## Discussion

### Genomic analyses of L7

In the present study, we complemented past descriptions of L7 [24, 26] with further information on its genomic profile and explored more deeply several cell wall loci by bioinformatic analyses, as well as the phenotypic consequences of a stop-gain mutation in the *mmaA3* gene of L7. At the genome level, we determined the number of L7-specific SNPs to be around 800, similar to a previous estimation [24]. This is higher than the number of lineage SNPs previously identified by a similar analysis of the six major *M. tuberculosis* lineages (between 124 and 698 SNPs, based on tracking each modern lineage back to the common modern origin), reflecting the shallow phylogeny represented by the limited set of relatively recently diverged strains that define L7. The overall pattern of L7 SNPs is similar to that documented for the major lineages [11] (dN/dS=0.54–0.65), generating a total of 482 non-synonymous mutations with a dN/dS ratio of 0.64. Although our sift analysis suggested that most non-synonymous mutations of L7 were tolerated, with a predicted functional impact of 13%, this was much lower than the 44 % that has been estimated for the six major *M. tuberculosis* lineages. However, much of that difference could be explained by a possible low specificity of the sift prediction methodology [52, 70]. Furthermore, to overcome these limitations, future integration of our results with additional experimental data on L7 could provide a more robust set of predicted functional variants [71].

Our bioinformatics results identified multiple mutations in the biosynthesis pathways of PGL/PDIM and mycolic acids (Table 2), mutations with potential consequences for the pathogenicity of *M. tuberculosis* L7. First, we analysed the PGL/PDIM locus and could confirm that L7 has the previously described 7 bp insertion in the *pks15/1* gene. *M. tuberculosis* strains that produce the potent immune modulator PGL contain this insertion leading to a single *pks15/1* ORF [64], such as strains of the hypervirulent W-Beijing family of L2 [72]. However, predicted functional SNPs in *pks15/1* and other genes of the PGL/PDIM locus still suggests a loss of PGL biosynthesis in L7, and the likely expression of variant forms of PDIM. A functional impairment of Rv2952, as could be the case in L7, could lead to loss of PDIM A and a possible accumulation of PDIM B [63]. However, the L7-specific SNP in Rv2952 cannot explain the downregulation (164-fold) of this methyltransferase shown in a comparative proteomic study of L7 versus *M. tuberculosis* L4 [26]. Altogether, it is possible that L7 strains have an impairment in the synthesis of the dimycoserosate-based lipids that could have an impact on their virulence and survival, in comparison to other lineages of *M. tuberculosis *.

The second gene cluster that drew our attention was the MymA (mycobacterial monooxygenase) locus that is involved in mycolic acid biosynthesis [65]. The VirS transcription factor (Rv3082c) has been shown to activate expression of the downstream *mymA* locus (Rv3083–Rv3089) in a kinase-dependent manner regulated by the adjacent PknK (Rv3080c). It has also been shown that the promotor of the *mymA* operon is induced in macrophages and upon exposure to acidic pH [65]. In addition, induction of the *mymA* operon by VirS is important for maintaining an appropriate cell envelope structure of *M. tuberculosis* [65], as well as required for maintaining the appropriate mycolic acid composition and permeability of the cell envelope on its exposure to acidic pH. Deletion of *virS* or *mymA* in *M. tuberculosis* H_37_Rv resulted in pleiotropic changes to the cell wall and reduced virulence in a guinea pig model of infection [73]. The results of our bioinformatics analysis concluded that L7 has an intact VirS but that mutations in the MymA locus and in associated proteins may have a regulatory impact on mycolic acid biosynthesis. In particular, the truncation of LipR (due to a stop-gain mutation; Table 2) may have a significant effect. A study [74] exploring LipR function in the mouse model suggested that this esterase may act as an immune modulator to inhibit the secretion of pro-inflammatory cytokines and consequently help avoid cidal host immune responses. However, further studies are required to understand what effects the mutations in the MymA locus of L7 may have on its ability to regulate mycolic acid composition and maintain cell envelope integrity, in comparison to other lineages.

While mutations in the MymA locus of *M. tuberculosis* L7 may have a regulatory impact on mycolic acid biosynthesis, the genes involved in the fatty/mycolic acid biosynthesis are all associated with the FAS I/II cycles. These two cycles are required for the biosynthesis of mycolic acid precursors of *M. tuberculosis *, as well as for their different chemical modifications (reviewed by PaweŁczyk and Kremer [36]). Nearly all genes involved in elongation and modification of the meromycolates in the FAS I/II pathways have been characterized, of which several are found in the *hadABC* and *mmaA1–4* gene clusters (Fig. 2a). Our screening for L7-specific mutations among 26 characterized FAS I/II genes [36] identified only one mutation with a predicted functional impact – the stop-gain mutation in the *mmaA3* gene. It can be noted that in a comparative proteomic study of *M. tuberculosis* L4 and L7, the *mmaA3* gene showed an 11-fold down-regulation in L7 [26].

### L7 lacks methoxy-mycolates

The methoxy-mycolic acid synthase (MmaA3) is responsible for *O*-methylation of mycolic acids, a modification in the biosynthesis of meromycolic acids in FAS II, leading to mMAs [36]. In the present study, we have shown evidence that the stop-gain mutation in *mmaA3* leads to a truncated (Fig. 2b) and an inactive (Fig. 3) MmaA3 enzyme. We found that all examined wild-type strains of L7 lacked mMA, while it was present in the investigated strains of *M. tuberculosis* L3 and L4. Earlier studies determined that while keto-MA and α-MA are present in most mycobacterial species, mMA appears, with a few exceptions, to be present only in pathogenic species and primarily in slow-growing mycobacteria, including all species of MTBC [59]. To the best of our knowledge, no other lineage of *M. tuberculosis sensu stricto* has been reported to be naturally deficient in mMA and our screening of nearly 5000 *M *. *tuberculosis* genomes of lineages other than L7 identified only two strains with potential functional defects of MmaA3. Hence, it can be proposed that this described phenotype of L7 – the absence of mMA in its cell envelope – defines this lineage of *M. tuberculosis *.

### Phenotypic effects due to methoxy-mycolate deficiency

Consequences of the deficiency in mMA were seen by TEM at the cell-level in 7H9 liquid medium. We concluded that cells of wild-type L7 had significantly reduced cording features as compared to MmaA3-complemented cells of L7 (Fig. 4). These changes could be a direct result of alterations in the physicochemical properties of the cell wall generated by altered composition or folding of the different mycolic acids [75], or secondary consequences associated with recruitment of a different complement of other cell wall components. Our lipid analysis did not include the extractable trehalose monomycolate (TMM) and trehalose dimycolate (TDM), but an absence of mMA among the mycolic acids attached to the AGP complex should also mean a deficiency of mMA in TMM and TDM as all mycolic acid residues originate from the same biosynthetic pathways [76]. Therefore, the TDM (or the cord factor) of L7 strains will only be composed of keto-MAs and α-MAs, and such alteration may contribute to changes in the cell wall structure and ability to form cords.

The change in cording seen by TEM may explain the differences observed when these strains were cultured in a biofilm assay using detergent-free medium. Biofilm assays of the two L7 strains showed that *mmaA3* complementation induced growth of macroscopic knotted cellular clusters; it is likely that *mmaA3* complementation allowed these strains to cord, aggregate, more effectively. The lack of a functional Mma*A3* may lead to reduction in cording and, thus, biofilm formation, which has been shown to be important in pathogenesis and disease *in vivo* [77]. In addition to changes in cording, short-chain mycolic acids have been shown to be important in the development of mycobacterial biofilms with mycobacteria embedded in a mMA-containing extracellular matrix [78].

Structural changes seen at the cellular level may also explain the distinct difference in colony morphology that we observed between wild-type and MmaA3-complemented strains of L7, when cultured on defined 7H11 medium (Fig. 6a). While the colony morphology of the complemented L7 strains resembled the classical morphological features of *M. tuberculosis *, the colonies of wild-type L7 strains had a smaller appearance, usually raised from the solid agar surface in a mushroom-shaped structure.

The above-described features of L7 are reminiscent of morphological changes and cording capacity reported for experimental mutation of genes that are responsible for cyclo-propanation and hydroxylation of mycolic acids, including the *mmaA1–4* genes. A deletion of *pcaA* generated a mutant with reduced cording [79] and a mutant deleted for *mmaA1–4*, *pcaA* and *cmaA2* showed smaller colonies and a smooth, ‘donut’ morphology when grown on 7H10 agar [80], in fact, a very similar phenotype as seen here for L7. This morphology has also been described for the single *mmaA4* mutant [81], located downstream of *mmaA3* (Fig. 2a), that was explained by the lack of oxygenated mycolates. In fact, the same authors also created a single *mmaA3* mutant of *M. tuberculosis* H_37_Rv that, when grown on 7H10 agar medium, resulted in a mixture of smooth and rough colony morphologies. However, due to this indistinct result, their subsequent work focused on the *mmaA4* mutant only [81]. A more recent study by Slama *et al*. [37] investigated *hadC* (*Rv0637*), a gene responsible for chain elongation in the FAS II cycle, and found that its deletion from *M. tuberculosis* H_37_Rv caused a significant reduction of oxygenated mycolic acids, with a loss of cording phenotype and change of colony morphology. The authors also showed that the same phenotype was expressed by the attenuated strain *M. tuberculosis* H_37_Ra and it was explained by a mutation in the *hadC* gene [37]. All these findings show that experimental disruption in the biosynthesis of mycolic acids, and especially oxygenated mycolates, cause phenotypic changes like those seen in *M. tuberculosis* L7 that is naturally mutated in the *mmaA3* gene of the FAS II cycle.

### *M. bovis* bacillus Calmette–Guérin (BCG) and methoxy-mycolates

Interestingly, a phylogenetic branch of *M. bovis* BCG that comprises BCG Danish and BCG Pasteur also has a defect in the biosynthesis of mMA [82]. As highlighted in Fig. 2(b), a non-synonymous SNP in the *mmaA3* gene (G98D) affects the conserved SAM binding site of MmaA3 and abolishes its enzymatic activity in BCG strains of that branch, while BCG strains that do not carry this mutation, including BCG Russia and BCG Tokyo, synthesize mMA. Behr *et al*. [82] have suggested that the deficiency of mMA is considered to reflect the differences in immunogenicity among BCG strains. To explore the impact of mMA further, Hayashi *et al*. [83] measured immune-stimulating activities of BCG sub-strains and investigated correlations between genetic background and immunological activities of the strains *in vitro*. They concluded that the phylogenetic branch of BCG strains that synthesize mMA exhibit a higher synergistic effect with cytokines on the production of nitric oxide and inflammatory cytokines and they suggested that BCG strains of this branch may confer better protection against TB and could be the better BCG vaccines currently used. In addition to these *in vitro* findings, BCG vaccine trials have shown differences in protective efficacy of BCG vaccines that possess or lack mMA, with BCG vaccines containing mMA considered more immunogenic. Behr *et al*. concluded that the ability of BCG strains to synthesize mMAs is likely but one of a number of determinants of how BCG vaccines behave *in vivo* [82].

### Conclusion

We conclude that the inability of *M. tuberculosis* L7 to produce mMA has an impact on cell wall structure and cording. The formation of serpentine cords, a morphology that was first noted by Robert Koch and later defined as cording, is correlated with the virulence of *M. tuberculosis* [79], with multiple further studies linking modification of cell wall structure to virulence in *M. tuberculosis* [37, 78, 81, 84–87]. However, whether and how the mMA defect in L7 may influence host–pathogen interaction remains to be shown.

While the limited number of strains identified from L7 precludes the large-scale population studies required for identification of epidemiological associations, the findings reported in the present study make an important contribution to our overall understanding of genetic diversity of *M. tuberculosis *. Comparison of lineages that have been successful in infecting a large proportion of the global population with those that have been restricted to small groups has the potential to provide insights into the fundamental biology of host–pathogen interactions and into the history of human societies.

## Supplementary Data

Supplementary material 1

Supplementary material 2
