# Corrigendum: Lack of methoxy-mycolates characterizes the geographically restricted lineage 7 of Mycobacterium tuberculosis complex

**DOI:** 10.1099/mgen.0.001226

**Published:** 2024-03-28

**Authors:** Elena Hailu, Daire Cantillon, Carlos Madrazo, Graham Rose, Paul R. Wheeler, Paul Golby, Bethlehem Adnew, Sebastien Gagneux, Abraham Aseffa, Stephen V. Gordon, Iñaki Comas, Douglas B. Young, Simon J. Waddell, Gerald Larrouy-Maumus, Stefan Berg

**Affiliations:** 1 Armauer Hansen Research Institute, Addis Ababa, Ethiopia; 2 Brighton and Sussex Centre for Global Health Research, Department of Global Health and Infection, Brighton and Sussex Medical School, University of Sussex, Falmer, UK; 3 Biomedicine Institute of Valencia, Spanish Research Council (IBV-CSIC), Valencia, Spain; 4 Francis Crick Institute, London, UK; 5 Animal and Plant Health Agency, Weybridge, UK; 6 Swiss Tropical and Public Health Institute, Allschwil, Switzerland; 7 University of Basel, Basel, Switzerland; 8 School of Veterinary Medicine, University College Dublin, Dublin, Ireland; 9 MRC Centre for Molecular Bacteriology and Infection, Imperial College London, London, UK

In the published version of this article the funding information was incorrectly listed as

This study was supported by the MRC Confidence in Concept Fund and the ISSF Wellcome Trust grants 105603/Z/14/Z (G.L- M) and 204833/Z/16/Z (S.J.W). E.H., A.A. and S.J.W. would like to acknowledge funding from the Association of Physicians of Great Britain and Ireland Links with Developing Countries Scheme, and the Human Hereditary and Health in Africa (H3Africa) ‘TBGEN: an integrated approach to unravelling susceptibility to tuberculosis in Africa’ [H3A/18/003]. H3Africa is managed by the Science for Africa (SFA) Foundation in partnership with the Wellcome Trust, the NIH (National Institutes of Health) and African Society of Human genetics (AfSHG). The views expressed herein are those of the authors and not necessarily those of the SFA Foundation and its partners.

The funding should have been listed as:

This study was supported by the Wellcome Trust grant 075833/A/04/Z (D.B.Y), the MRC Confidence in Concept Fund, and the ISSF Wellcome Trust grants 105603/Z/14/Z (G.L- M) and 204833/Z/16/Z (S.J.W). E.H., A.A. and S.J.W. would like to acknowledge funding from the Association of Physicians of Great Britain and Ireland Links with Developing Countries Scheme, and the Human Hereditary and Health in Africa (H3Africa) ‘TBGEN: an integrated approach to unravelling susceptibility to tuberculosis in Africa’ [H3A/18/003]. H3Africa is managed by the Science for Africa (SFA) Foundation in partnership with the Wellcome Trust, the NIH (National Institutes of Health) and African Society of Human genetics (AfSHG). The views expressed herein are those of the authors and not necessarily those of the SFA Foundation and its partners.

The authors apologise for any inconvenience caused.

