# Supplementary material for: Lack of methoxy-mycolates characterizes the geographically restricted lineage 7 of Mycobacterium tuberculosis complex
Source: Microb Genom. 2023 May 12;9(5):mgen001011. doi: 10.1099/mgen.0.001011 (PMC10272862; doi:10.1099/mgen.0.001011)
Supplement: Supplementary material 1 [file mgen-9-1011-s001.pdf]

Supplementary Material

Content

Supplementary results

Supplementary discussion

Supplementing Table S4

Supplementary Figures S1-S6

Supplementary results

Colony morphology of Lineage 7 varies on different Middlebrook media

The Modified 7H11 agar medium used in this study was developed in the 1970ies (Gallagher & Horwill, 1977) and is used on a routine basis in our laboratory for culturing of mycobacteria. Its composition is listed in Supplementary Table S4. In comparison to the standard Middlebrook 7H11 agar medium supplemented with OADC (Oleic acid, Albumin, Dextrose, and Catalase) only, the modified version contains also additional Malachite Green, Ovine Blood, Adult Bovine Serum, as well as several anti-microbial and anti-fungi drugs, making it highly selective for mycobacterial growth. No glycerol or Tween 80 is added to the Modified 7H11 agar medium.

To seek clues to whether a specific component of Modified 7H11 would be directly responsible for the raised and crumpled colony morphology of the L7 strains, we cultured two L7 wild-type strains (BTBH-568 and BTBH-444) on standard Middlebrook 7H11 (BD) agar supplemented with OADC only (no drugs added; Supplementary Table S4). As when grown on Modified 7H11 agar, a raised crumpled colony morphology could still be observed for these two strains, while the corresponding constructs complemented with *mmaA3* were presented with an eugonic colony morphology (Fig. S4B). We could therefore conclude that the difference in composition between Modified 7H11 and standard 7H11 agar media had no impact on the difference in colony morphology seen between wild-type L7 strains and ditto complemented with *mmaA3*.

Interestingly, when culturing the same L7 strains (BTBH-568 and BTBH-444) on 7H10 agar medium (supplemented with OADC only), we observed that the colonies of the wild-type strains had an eugonic morphology instead of the raised crumpled morphology seen on 7H11 agar and the same eugonic morphology was observed when these L7 strains were complemented with the empty vector (EV) control and with *mmaA3* (Fig. S4A). However, the colonies grew larger when these L7 strains were complemented with *mmaA3*. We conclude that growth on 7H10 agar do not trigger a raised crumpled morphology in the L7 wild-type strains, but growth was still compromised due to lack of methoxy-mycolates, as smaller eugonic colonies was observed as compared to the morphology of strains

complemented with *mmaA3*. As a comparison between L7 and L3, the three corresponding L3 strains of BTBH-273 (wild-type/*mmaA3*/EV control) showed no significant difference in morphology, neither on Modified 7H11 (Fig. 6), standard 7H11 agar, or standard 7H10 agar (data not shown).

To explain the differences in morphology seen for L7 on these media we explored their contents. The base powders of Middlebrook 7H10 and Middlebrook 7H11 (both provided by BD) had the same composition except for the addition of 0.1% pancreatic digest of casein to Middlebrook 7H11 (Supplementary Table S4). Therefore, we propose that the added casein digest is the component in 7H11 that has a direct or indirect impact on the colony morphology of L7 strains leading to a raised crumpled structure on all tested 7H11 media types.

The impact of glycerol as a growth supplement

Independent of which Middlebrook medium (7H10 or 7H11 based) that was used in this study for comparing colony morphology of wild-type and complemented L7 strains, the colony size was consistently smaller for wild-type strains of L7 (mMA absent) as compared to L7 strains complemented with *mmaA3* (mMA present). However, this difference could be reduced by adding glycerol to the growth medium, and especially when grown on 7H10. Fig. S4A shows the effect on morphology of BTBH-568 (L7) when a titration with glycerol at concentrations 0%, 0.5%, and 2.5% was performed on 7H10 agar. The colonies of all constructs (wild-type/*mmaA3*/EV control) increased significantly in size with increased glycerol concentration but complementation with *mmaA3* had highest impact. These morphological effects were similar on both 7H10 and 7H11 (Supplementary Fig. S4A and S4B).

To investigate whether the effect of glycerol seen on solid medium could be observed also in liquid culture, we supplemented 7H9 liquid medium with 2% glycerol and performed TEM to explore whether it had an influence on L7 cell morphology. Indeed, the wild-type strain BTBH-568 (L7), which showed an abnormal phenotype when grown without glycerol (Fig. 3), displayed a cell morphology similar to reference strain $H_{37}Rv$ as well as BTBH-568 complemented with *mmaA3*, when grown with glycerol (Supplementary Fig. S5).

Supplementary discussion

The effect of glycerol and casein on L7 cell structure and colony morphology

Our experiments show that different media compositions (7H11 and 7H10 and supplementations thereof) influenced on the morphology, with glycerol and casein-digest being two media components that seemed to have a visual impact on the morphology (Supplementary Fig. S4A and S4B). An increased glycerol concentration in the agar medium compensated for the morphological artefact of the wild-type L7 strains, leading to larger colonies with eugonic features of the colonies. Interestingly, at cell-level the TEM results also indicated an effect when glycerol was added to the media (Supplementary Fig. S5). The wild-type strain BTBH-568 of L7 showed a reverted cell morphology in the presence of glycerol. The reason for this may be that supplementation with glycerol as a carbon source influence the cell metabolism of the pathogen with altered cell-wall composition as a consequence, and/or because glycerol is taken up from the growth medium and directly incorporated into the cell envelope with structural effects. The same explanations can be proposed for the morphological difference when a pancreatic digest of casein was supplemented to 7H11 agar media. Casein is a protein that has high proline content and a high degree of hydrophobic amino acids, and a digest of casein may provide the culture with peptides that have a direct or indirect effect on the cell and colony morphology of L7. It is also possible that glycerol can quench the effect seen by the casein-digest, through interaction with the same.

In conclusion, it can be proposed that the addition of glycerol or the absence of a casein-digest can restore the L7 morphology to an eugonic feature when grown on these media *in vitro*; however, these findings cannot clarify the possible role that mMA has in host-pathogen interactions and as a virulence factor *in vivo*.

Supplementing Table S4. Composition of Middlebrook media used in this study. All amounts are documented in grams (or millilitres if stated) per Litre of distilled water.

Component	7H9	7H10 (BD)	7H11 (BD)	Modified 7H11(BD)
Base:				
Magnesium Sulfate	0.05	0.025	0.050	0.050
Ferric Ammonium Citrate	0.04	0.04	0.04	0.04
Sodium Citrate	0.1	0.4	0.4	0.4
Pyridoxine	0.001	0.001	0.001	0.001
Ammonium Sulfate	0.5	0.5	0.5	0.5
Zinc Sulfate	0.001	0.001	0.001	0.001
Monosodium Glutamate	0.5	0.5	0.5	0.5
Copper Sulfate	0.001	0.001	0.001	0.001
Disodium Phosphate	2.5	1.5	1.5	1.5
Biotin	0.0005	0.0005	0.0005	0.0005
Monopotassium Phosphate	1.0	1.5	1.5	1.5
Calcium Chloride	0.0005	0.0005	0.0005	0.0005
Agar	-	17.0	13.5	13.5
Malachite Green (in base)	-	0.00025	0.00025	0.00025
Pancreatic digest of Casein	-	-	1.0	1.0
Additives:				
OADC Enrichment (100.0 ml):				
Sodium Chloride	*0.85*	*0.85*	*0.85*	*0.85*
Oleic acid (ml)	-		*0.05*	*0.05*
Bovine Albumin V	*5.0*	*5.0*	*5.0*	*5.0*
Glucose / Dextrose	*2.0*	*2.0*	*2.0*	*2.0*
Catalase	*0.003*	*0.004*	*0.004*	*0.004*
Glycerol* / Tween 80	- / 0.5	5.0 / -	5.0 / -	- / -
Malachite Green (additive)		-	-	0.006
Heated Adult Bovine Serum (ml)		-	-	100
Fungizone		-	-	0.00005
Polymyxin B (IU)		-	-	200
Trimethoprim Lactate		-	-	0.01
Amoxicillin		-	-	0.1
Lysed Ovine Blood (Sterile) (ml)		-	-	2.5

* Glycerol concentration was titrated in some experiments of this study.

Supplementary Fig. S1. Conserved Domain analysis of selected proteins from three gene clusters discussed in this study (Table 2 and Fig. 2A). Positions of the mutated amino acids conserved in L7 are highlighted by arrows.

S1:A Rv3080c (pknK);

S1:B Rv3089 (fadD13);

S1:C Rv2931 (ppsA);

S1:D Rv2940c (mas);

S1:E Rv2946c (pks1);

S1:F Rv2947c (pks15);

S1:G Rv2952;

S1:H Rv2962c;

S1:I Rv0643c (mmaA3)

Supplementary Figure S1:A

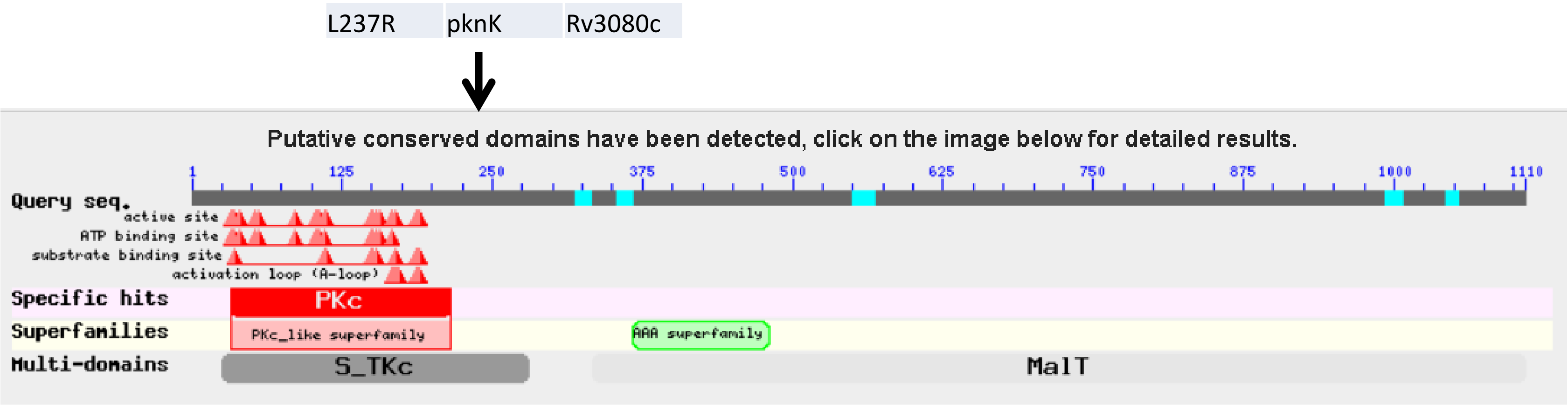

Supplementary Figure S1:B

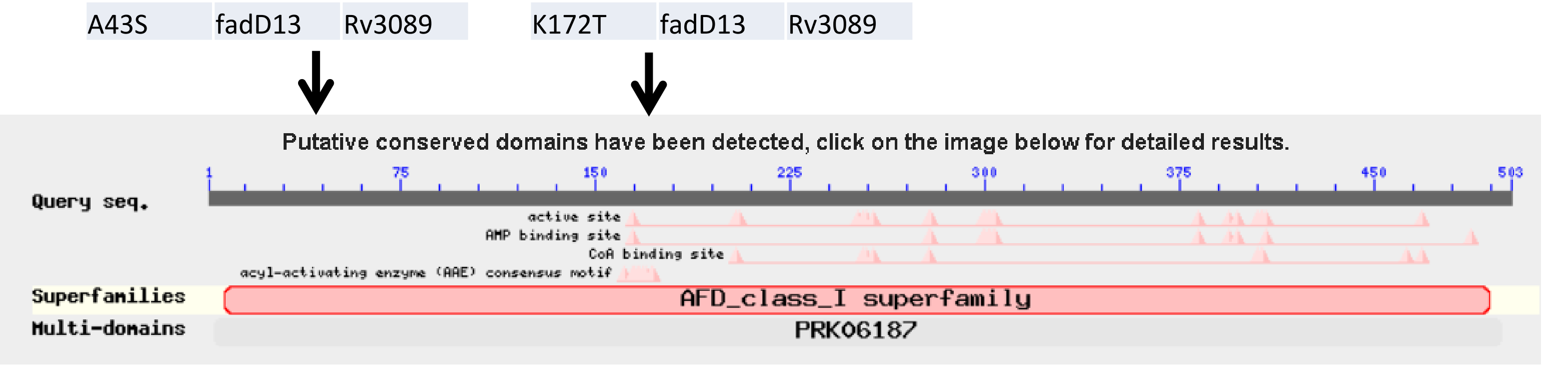

Supplementary Figure S1:C

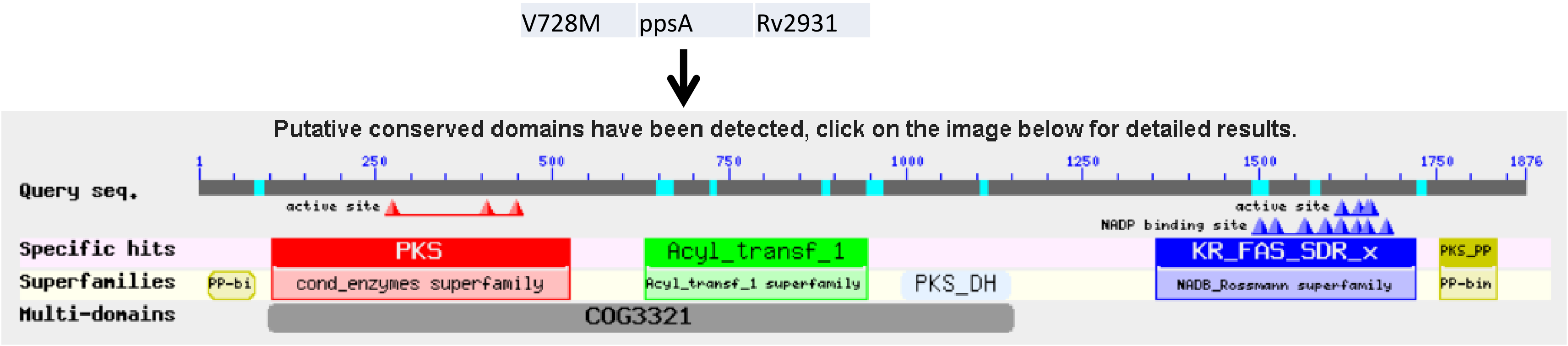

Supplementary Figure S1:D

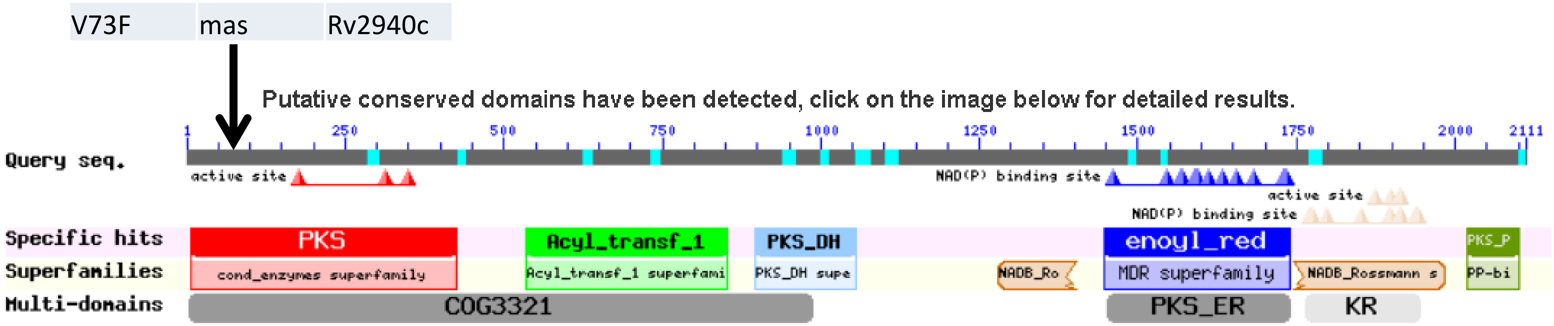

Supplementary Figure S1:E

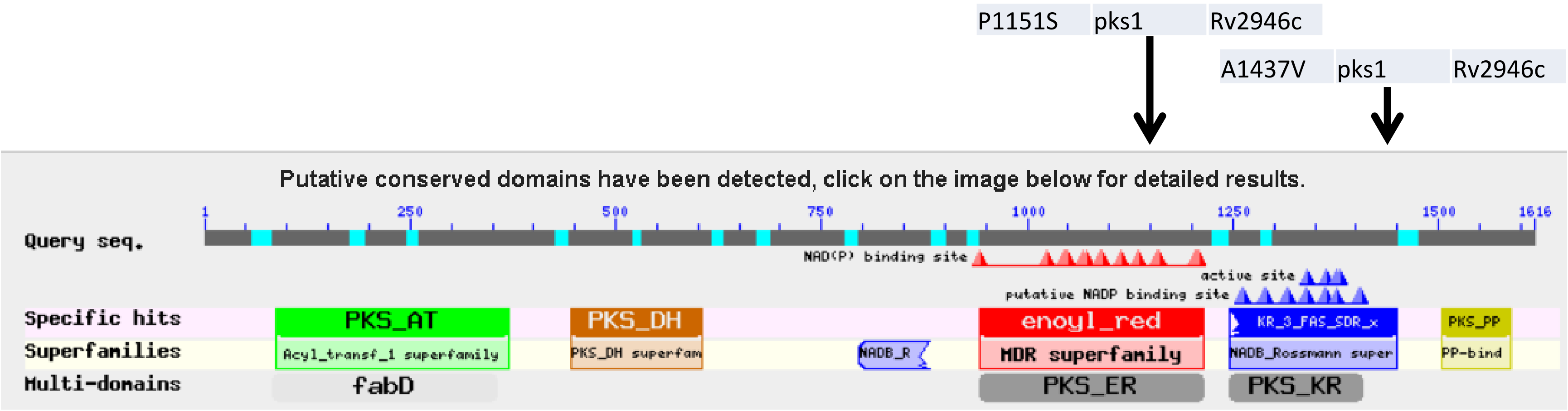

Supplementary Figure S1:F

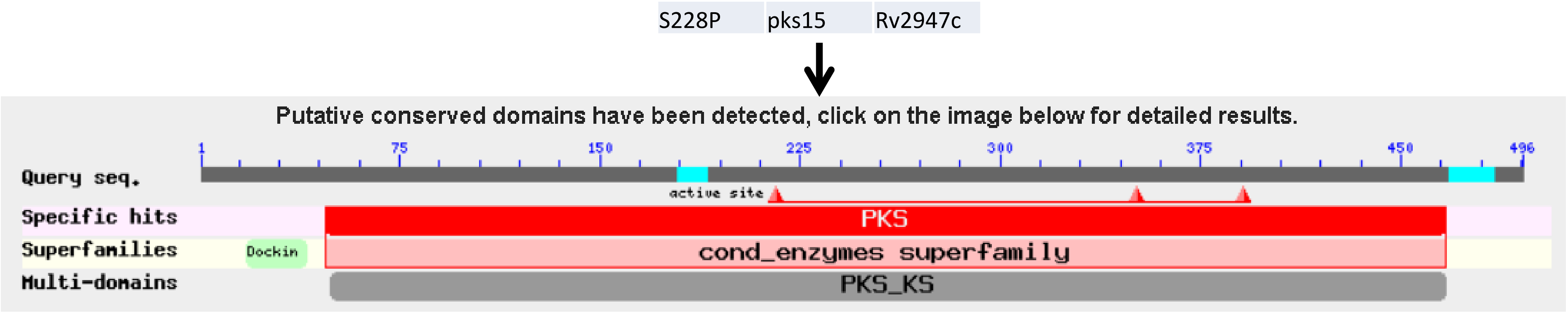

Supplementary Figure S1:G

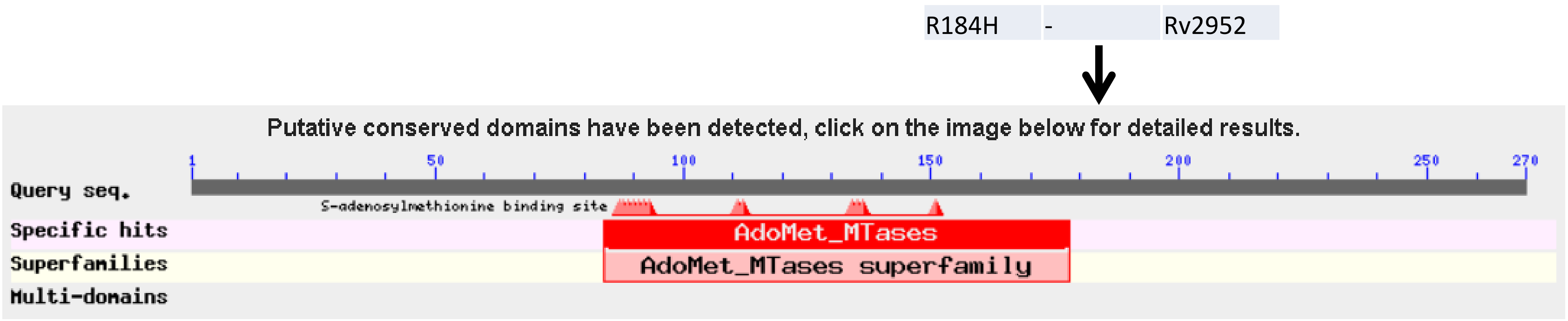

Supplementary Figure S1:H

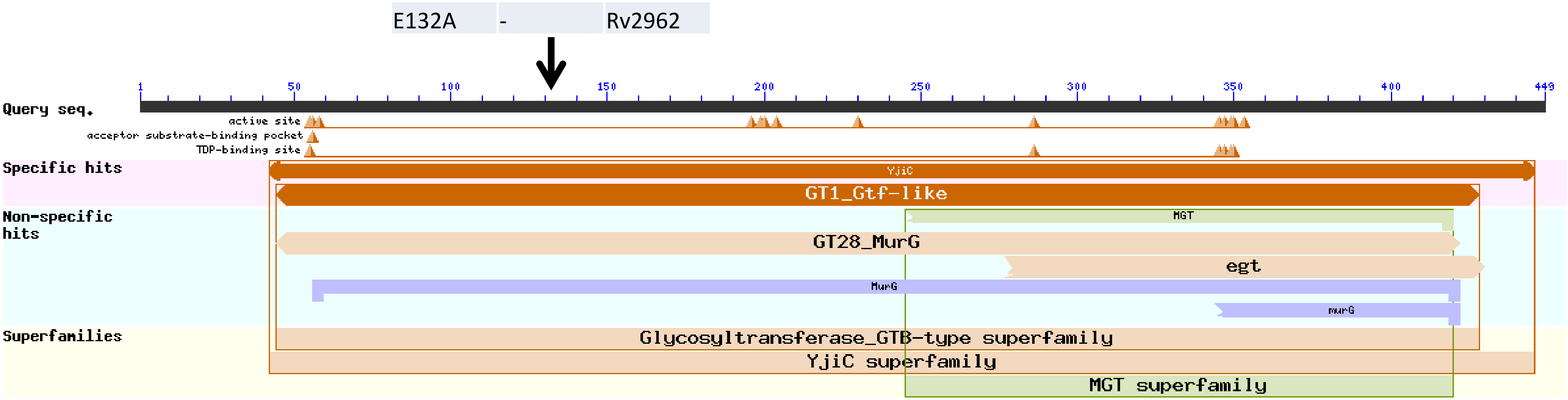

Supplementary Figure S1:I

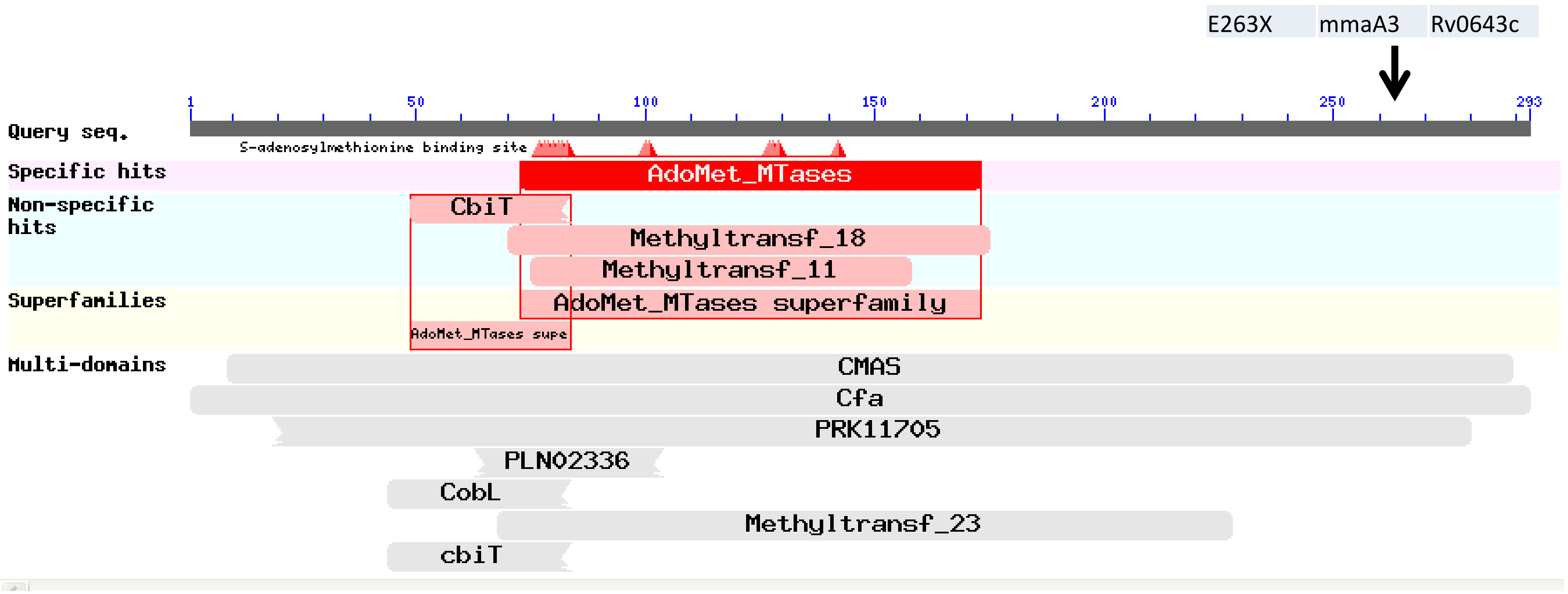

Supplementary Figure S2

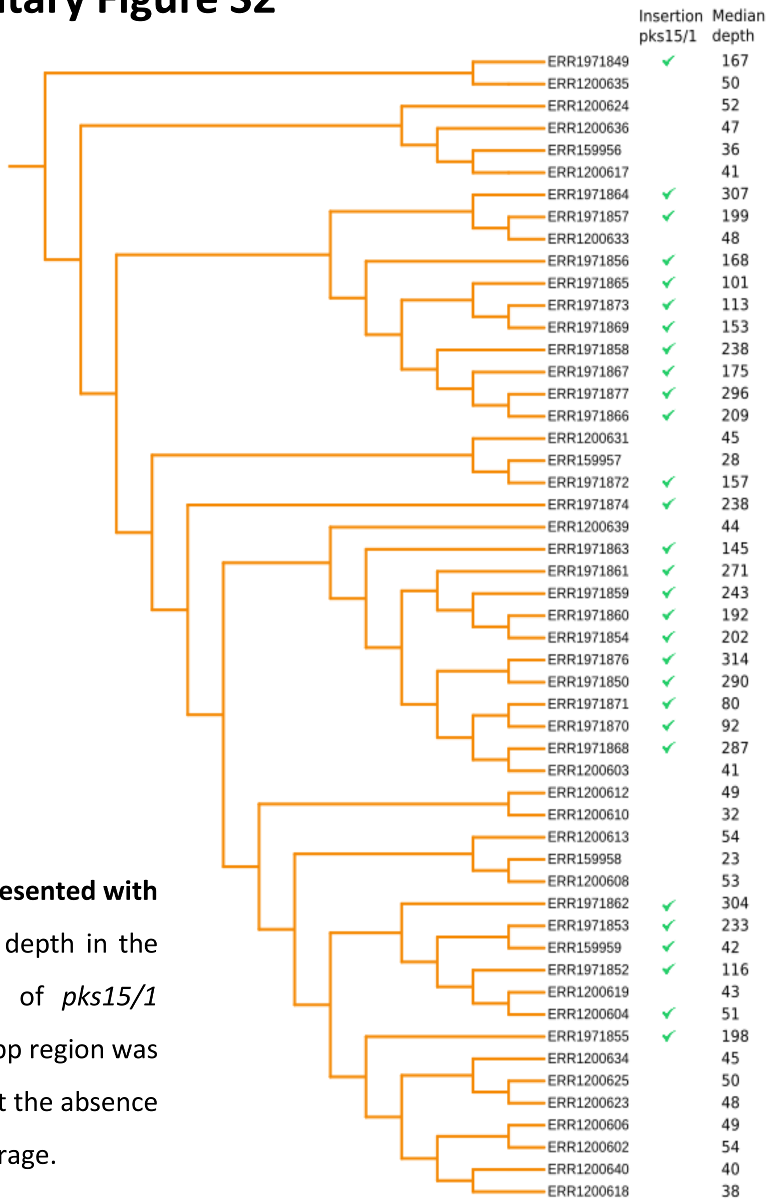

Supplementary Fig. S2. Phylogeny of 52 Lineage 7 isolates presented with median sequence coverage of respective genome. Median depth in the genomes where we detected the 7bp region (Insertion) of *pks15/1* was 192 (range 42-314) while, in those genomes where the 7bp region was absent, it was 45 (range 23-54). The data strongly suggest that the absence of these 7bp in some genomes was due to low sequence coverage.

Supplementary Figure S3

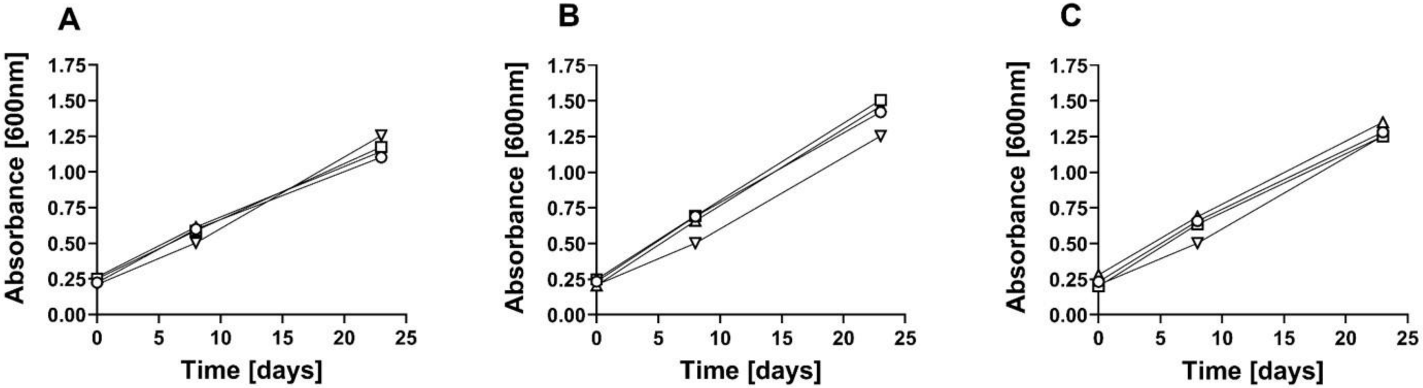

Supplementary Fig. S3. Growth curves of *Mycobacterium tuberculosis*. Growth of three constructs, WT (○), WT complemented with *mmaA3* (□), and WT complemented with EV control (Δ) of: A. Strain BTBH-273 (L3), B. Strain BTB-444 (L7), and C. Strain BTBH-568 (L7) were compared with reference strain *M. tuberculosis* $H_{37}Rv$ (∇) of L4. Strains were inoculated in 7H9 broth and incubated at 37 °C. OD^{600} were measured at Day 8 and Day 23. Representative growth curves from single biological replicates are displayed.

Supplementary Figure S4

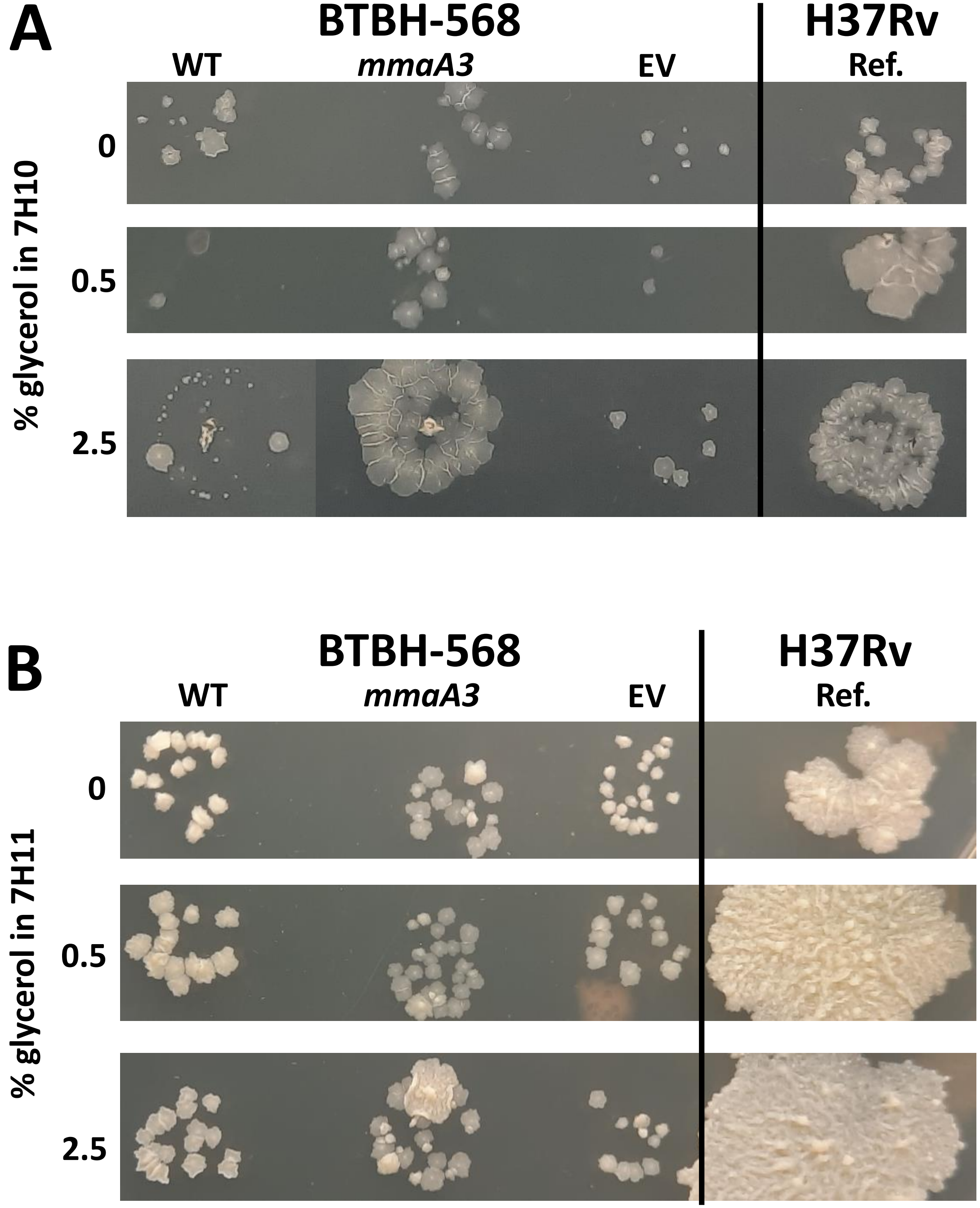

Supplementary Fig. S4. Colony morphology of *Mycobacterium tuberculosis* isolates grown on Middlebrook 7H10 and 7H11 solid media. Colony morphology shown for three different constructs (WT, WT complemented with *mmaA3*, and WT complemented with EV control) of BTBH-568 (L7), as well as for the reference strain *Mycobacterium tuberculosis* $H_{37}Rv$ when grown on Middlebrook 7H10 (A) and 7H11 (B) at three different glycerol concentrations (0%, 0.5%, and 2.5%). The difference seen between 7H10 and 7H11 can be associated with the presence of 0.1% Pancreatic digest of Casein in 7H11 (Supplementary Table S4). The corresponding results for the three constructs of BTBH-444 (L7) was very similar as to those of BTBH-568 (L7) (data not shown).

Supplementary Figure S5

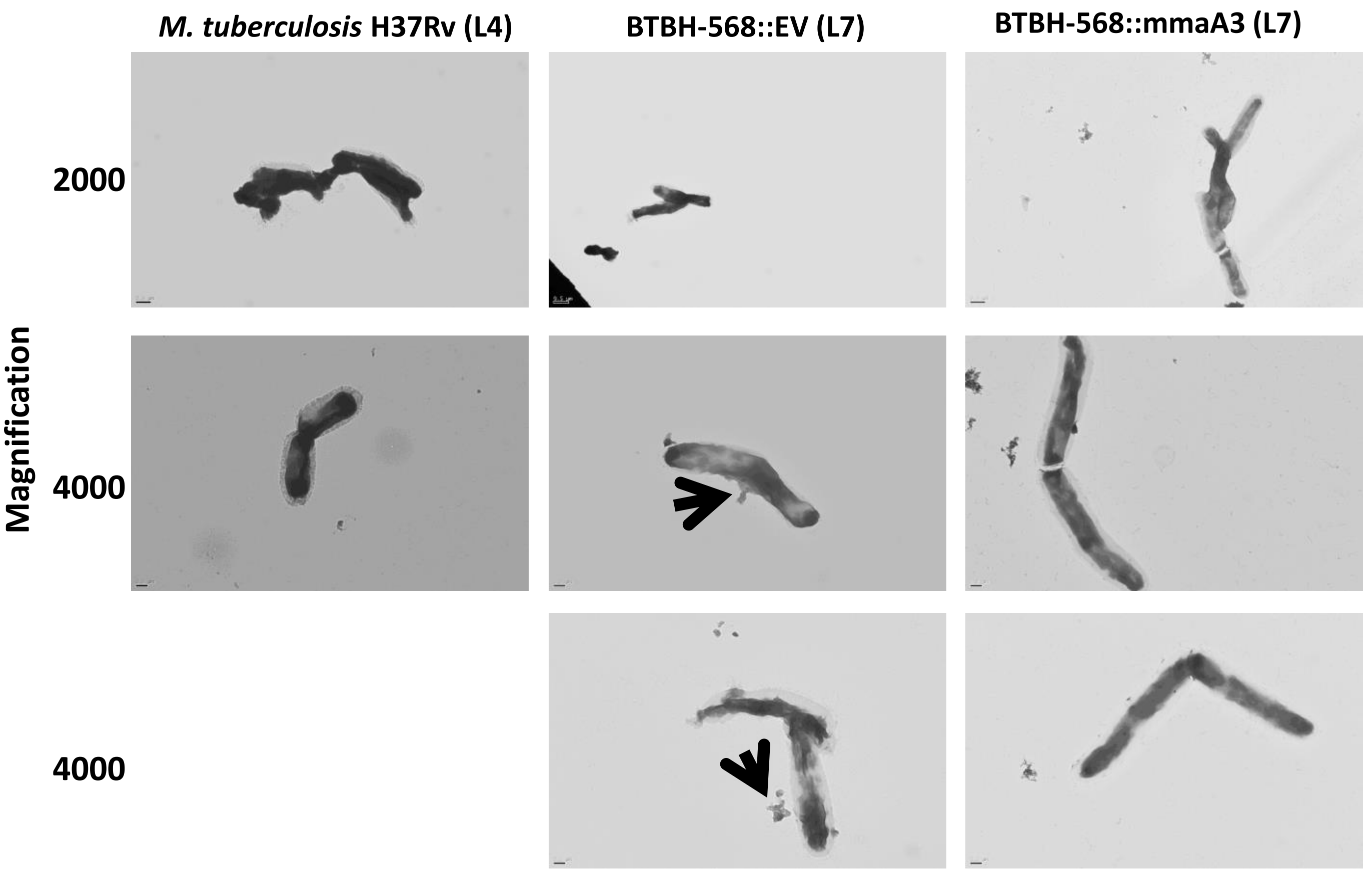

Supplementary Fig. S5. Transmission Electron Microscope structures of Lineage 7 grown in 7H9 broth with glycerol. L7 strain BTBH-568 complemented with EV control and *mmaA3*, respectively, was used for this experiment in parallel to the reference strain *Mycobacterium tuberculosis* $H_{37}Rv$. A Jeol 120 EX transmission electron microscope was used for analysis and images were acquired at 1,200x - 4,000x magnification. Arrows highlight reduced cording phenotype of L7, suggesting an impaired cell envelope.

Supplementary Figure S6

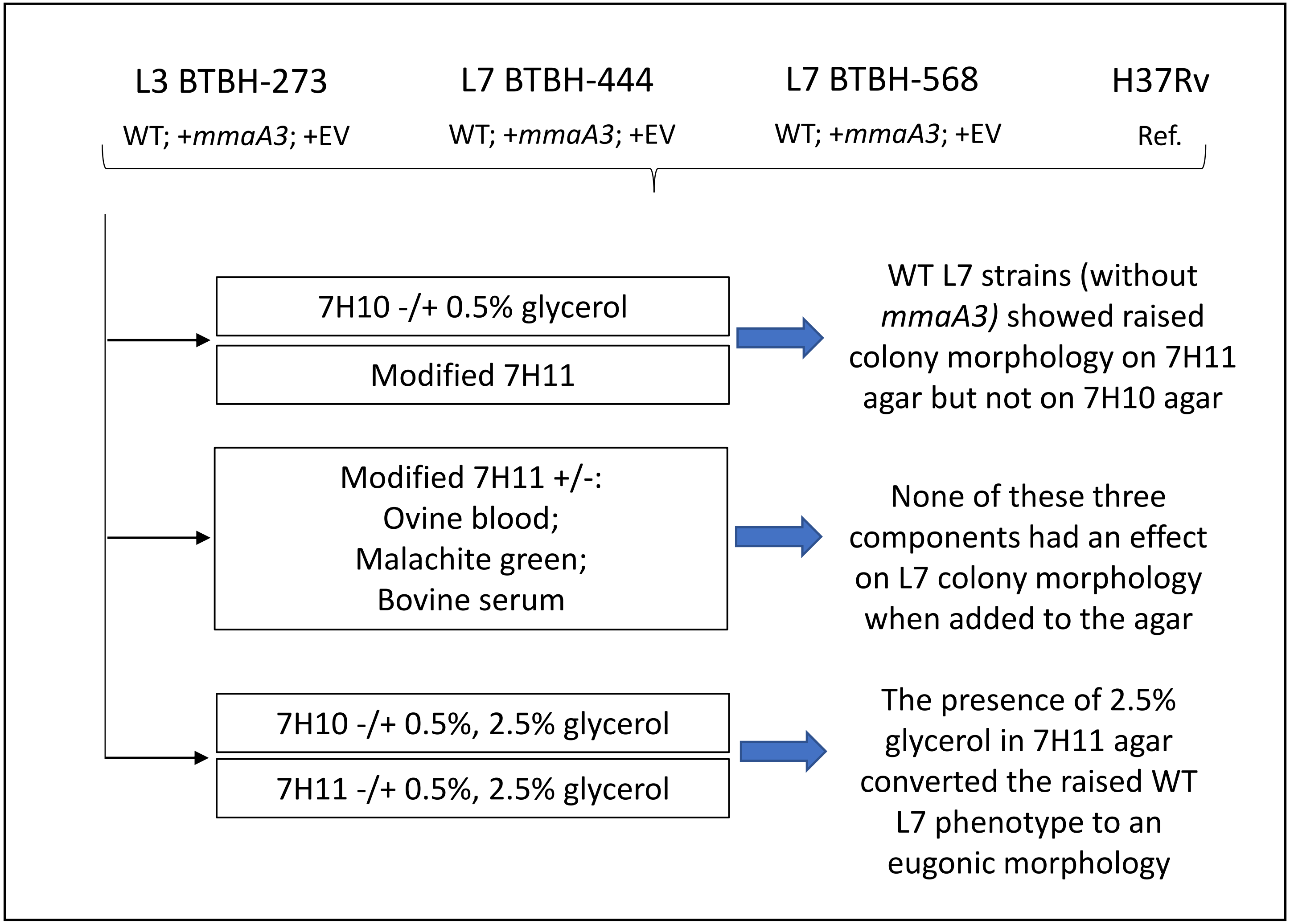

Supplementary Fig. S6. Workflow for L7 colony morphology experiments using *M. tuberculosis* L3 and L7 strains (WT, wildtype) and their complements with *mmaA3* and EV control, as well as reference strains *M. tuberculosis* $H_{37}Rv$.
